# Learning joint segmentation of tissues and brain lesions from task-specific hetero-modal domain-shifted datasets

**DOI:** 10.1016/j.media.2020.101862

**Published:** 2020-10-09

**Authors:** Reuben Dorent, Thomas Booth, Wenqi Li, Carole H. Sudre, Sina Kafiabadi, Jorge Cardoso, Sebastien Ourselin, Tom Vercauteren

**Affiliations:** a King’s College London, School of Biomedical Engineering & Imaging Sciences, St. Thomas’ Hospital, London, United Kingdom; b Department of Neuroradiology, King’s College Hospital NHS Foundation Trust, London, United Kingdom; c Dementia Research Centre, UCL Institute of Neurology, UCL, London, United Kingdom; d Department of Medical Physics, UCL, London, United Kingdom; e NVIDIA, Cambridge, United Kingdom

**Keywords:** Joint learning, Domain adaptation, Multi-Task learning, Multi-Modal

## Abstract

Brain tissue segmentation from multimodal MRI is a key building block of many neuroimaging analysis pipelines. Established tissue segmentation approaches have, however, not been developed to cope with large anatomical changes resulting from pathology, such as white matter lesions or tumours, and often fail in these cases. In the meantime, with the advent of deep neural networks (DNNs), segmentation of brain lesions has matured significantly. However, few existing approaches allow for the joint segmentation of normal tissue and brain lesions. Developing a DNN for such a joint task is currently hampered by the fact that annotated datasets typically address only one specific task and rely on task-specific imaging protocols including a task-specific set of imaging modalities. In this work, we propose a novel approach to build a joint tissue and lesion segmentation model from aggregated task-specific hetero-modal domain-shifted and partially-annotated datasets. Starting from a variational formulation of the joint problem, we show how the expected risk can be decomposed and optimised empirically. We exploit an upper bound of the risk to deal with heterogeneous imaging modalities across datasets. To deal with potential domain shift, we integrated and tested three conventional techniques based on data augmentation, adversarial learning and pseudo-healthy generation. For each individual task, our joint approach reaches comparable performance to task-specific and fully-supervised models. The proposed framework is assessed on two different types of brain lesions: White matter lesions and gliomas. In the latter case, lacking a joint ground-truth for quantitative assessment purposes, we propose and use a novel clinically-relevant qualitative assessment methodology.

## 1 Introduction

Traditional approaches for tissue segmentation used in brain segmentation / parcellation software packages such as FSL (Jenkinson et al., 2012), SPM (Ashburner and Friston, 2000) or NiftySeg (Cardoso et al., 2015) are based on subject-specific optimisation. FSL and SPM fit a Gaussian Mixture Model to the MR intensities using either a Markov Random Field (MRF) or tissue prior probability maps as regularisation. Alternatively, multi-atlas methods rely on label propagation and fusion from multiple fully-annotated images, i.e. atlases, to the target image (Cardoso et al., 2015; Iglesias and Sabuncu, 2015). These methods typically require extensive pre-processing, e.g. skull stripping, correction of bias field and registration. They are also often time-consuming and are inherently only adapted for brains devoid of large anatomical changes induced by pathology, such as white matter lesions and brain tumours. Indeed, it has been shown that the presence of lesions can significantly distort any registration output (Sdika and Pelletier, 2009). Similarly, lesions introduce a bias in the MRF. This leads to a performance degradation in the presence of lesions for brain volume measurement (Battaglini et al., 2012) and any subsequent analysis.

While quantitative analysis is expected to play a key role in improving the diagnosis and follow-up evaluations of patients with brain lesions, current tools mostly focus on quantification of the lesions themselves and effectively discard contextual tissue information. Existing quantitative neuroimaging approaches allow the extraction of imaging biomarkers such as the largest diameter, volume, and count of the lesions. Such automatic segmentation of the lesions promises to speed up and improve the clinical decision-making process but more refined analysis would be feasible from tissue classification and region parcellation. In particular, brain atrophy at a global level (Popescu et al., 2013; Giorgio and De Stefano, 2013), at a cerebral level (Bermel and Bakshi, 2006), and, even more specifically, at the grey matter level (Geurts et al., 2012) have been correlated with the speed of disease progression and with physical disability (Fisniku et al., 2008). Consequently, atrophied tissue volumes in the presence of lesions are clinically relevant imaging markers (Dwyer et al., 2018). We believe that, although very few work have addressed this problem yet, a joint model for lesion and tissue segmentation is expected to bring significant clinical benefits. As representative exemplars of the technical challenges involved to build such joint models, we focus, in this work, on patients with white matter lesions or brain tumours.

**Fig. 1:**
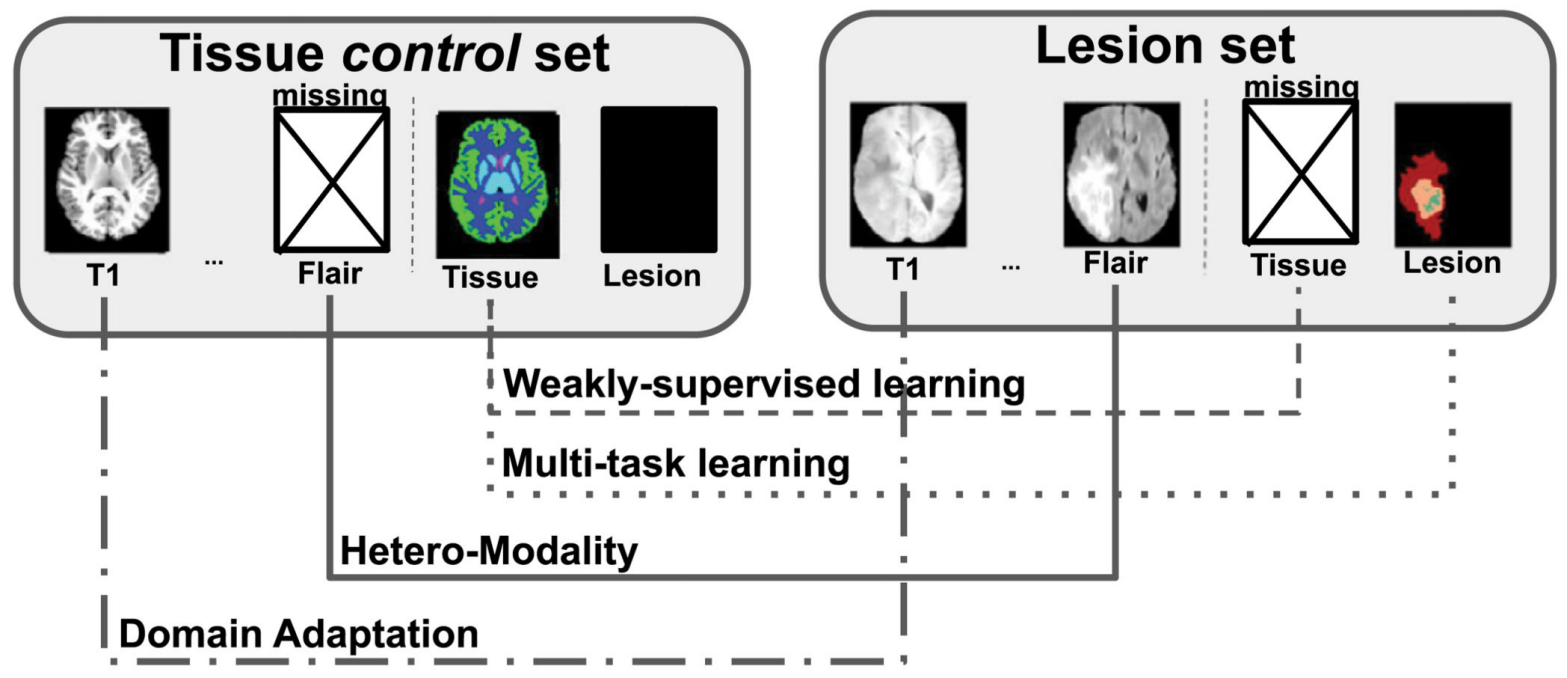
Tissue and lesion segmentation, a problem at the intersection of multiple branches of Machine Learning: Multi-Task Learning (tissue + lesion segmentation), Weakly-Supervised Learning (missing annotations), Hetero-Modality (missing modalities), Domain Adaptation (different acquisition protocols).

Deep Neural Networks (DNNs) have become the state-of-the-art for most segmentation tasks (Simpson et al., 2019) and one would now expect these to be able to jointly perform brain tissue and pathology segmentation. However, annotated databases required to train DNNs are usually dedicated to a single task (either brain tissue segmentation or pathology delineation). In addition, the information required for brain tissue or pathology segmentation may come from different scans, leading to hetero-modal (i.e. more than one set of input imaging sequences) datasets. While *T*_1_ -weighted images provide the best grey/white matter contrast for the delineation of anatomical tissue, *T*_2_ -weighted sequences are usually more sensitive to pathological changes (Bitar et al., 2006). Choice of the used sequence or combination of sequences may also differ across pathologies. *T*_2_ -weighted FLAIR images are often used for the assessment of white matter lesions (Maillard et al., 2013) while a combination of *T*_1_ contrast-enhanced (*T*_1_ c), *T*_2_ and FLAIR is often preferred for the characterisation of gliomas (Wen et al., 2010). Similarly, the scans may have been acquired with different magnetic resonance parameters leading to differences in resolution and contrast among databases. Consequently, the data distribution may differ between the datasets, i.e. the datasets may be domain-shifted. Given 1) the large amount of resources, time and expertise required to annotate medical images, 2) the varying imaging requirement to support each individual task, and 3) the availability of task-specific databases, it is unlikely that large databases for every joint problem, such as lesion and tissue segmentation, will become available. There is thus a need to exploit existing task-specific databases to address the joint problems. Learning a joint model from task-specific hetero-modal and domain-shifted datasets is nonetheless challenging. As shown in Fig. 1, this problem lies at the intersection of Multi-Task Learning (Zhang and Yang, 2017), Domain Adaptation (Ben-David et al., 2010; Zhao et al., 2019) and Weakly Supervised Learning (Oquab et al., 2015; Bilen and Vedaldi, 2016; Xu et al., 2014) with singularities making individual methods from these underpinning fields insufficient to address it completely, as explained in more depth in the related work section 2.

Our approach is rooted in all these sub-domains of deep learning. The main contributions are summarised as follows:

1. We propose a joint model that performs tissue and lesion segmentation as a unique joint task and thus exploits the interdependence between the lesion and tissue segmentation tasks. Starting from a variational formulation of the joint problem, we exploit the disjoint nature of the label sets to propose a practical decomposition of the joint loss, transforming the multi-class segmentation problem into a multi-task problem.
2. We introduce feature channel averaging across modalities to adapt existing networks for our hetero-modal problem.
3. We develop a new method to minimise the expected risk under the constraint of missing modalities. Under the assumption that the network is not affected by a potential domain shift, we show that the expected risk can be further decomposed and minimised via a tractable upper bound. To our knowledge, no such optimisation method for missing modalities in deep learning has been published before.
4. Given that, in practice, the heterogeneous task-specific datasets may have been acquired with different protocols, i.e. they are domain-shifted, we integrate several existing DA techniques in our framework. These methods are based on data augmentation and adversarial training, or pseudo-healthy brain generation.
5. We demonstrate the performance of our joint approach on two clinical use cases: White matter lesions and gliomas. Our method outperforms a fully-supervised model trained on a smaller fully-annotated dataset for white matter lesions. To assess the performance of the joint model for tissue and glioma segmentation, for which no ground-truth is available, we propose a new qualitative evaluation protocol based on the ASPECTS score (Barber et al., 2000). Higher accuracy is obtained compared to time-consuming pipelines that require to mask the lesions using manual annotations.
6. Experiments show that generating pseudo-healthy annotated scans outperforms the other DA techniques, even with very few pseudo-healthy annotated scans.

This work is a substantial extension of our conference paper (Dorent et al., 2019b). Improvements include: 1) Additional mathematical proofs; 2) integration and validation of three different domain adaptation techniques to cope with domain-shifted datasets; 3) new experiments on joint brain tissue and glioma segmentation; and 4) a new quantitative evaluation protocol for assessing tissue segmentation in the absence of ground-truth.

## 2 Related work

Multi-Task Learning (MTL) aims to perform several tasks simultaneously, on a single dataset, by extracting some form of common knowledge or representation and introducing a task-specific back-end. When relying on DNN for MTL, the first layers of the network are typically shared, while the last layers are trained for the different tasks (Ruder, 2017). MTL has been successfully applied to medical imaging for segmentation (Bragman et al., 2018; Moeskops et al., 2016) combined with other tasks such as detection (Saha et al., 2019) or classification (Chen et al., 2019; Le et al., 2019). The global loss function is a weighted sum of task-specific loss functions. Recently, Kendall and Gal (2017) proposed a Bayesian parameter-free method to estimate the MTL loss weights and Bragman et al. (2018) extended it to spatially adaptive task weighting and applied it to medical imaging. Although the aforementioned approaches generate different outputs from the same features, no direct interaction between the task-specific outputs is modelled in these techniques. While a joint tissue and lesion segmentation can be pursued in practice, a strong underpinning assumption is that the two outputs are conditionally independent. Consequently, these approaches do not address the problem of aggregating these outputs to generate a joint segmentation. Moreover, MTL approaches, such as (Moeskops et al., 2018; Roulet et al., 2019), do not provide any mechanism for dealing with heteromodal datasets or changes in imaging characteristics across task-specific databases.

Domain Adaptation (DA) is a solution for dealing with domain-shifted datasets, i.e. datasets acquired with different settings. A classical strategy consists in learning a domain-invariant feature representation of the data. Csurka (2017) proposed an extensive review of these methods in deep learning. Some DA approaches have been developed to tackle a specific and identified shift. For example, data augmentation has been used for shifts caused by different MR bias fields (Sudre et al., 2017) or the presence of motion artefacts (Shaw et al., 2019); Havaei et al. (2016) and Dorent et al. (2019a) proposed network architectures for dealing with missing modalities that encodes each modality into a shared modality-agnostic latent space. Recent studies have proposed to learn a mapping between healthy and decease scans, using Cy-cleGANs (Xia et al., 2019; Sun et al., 2018) or Variational Autoencoders (Chen and Konukoglu, 2018). Although these techniques have shown promising results, they are inherently limited to a specific type of shift. Combining causes of shift, for instance the presence/absence of lesions with different protocols of acquisition, remains an unsolved problem. In contrast, general DA approaches do not make assumptions about the nature of the shift. These methods aim to directly minimise the discrepancy between the feature distributions across the domains. Distribution dissimilarity can be assessed using correlation distances (Sun et al., 2016) or maximum mean discrepancy (Pan et al., 2011; Long et al., 2015). However, more recent techniques are mostly focused on adversarial methods, achieving promising results in medical imaging (Kamnitsas et al., 2017; Dou et al., 2018; Orbes-Arteaga et al., 2019). However, these methods are usually focus on solving a single task across domain.

Weakly-supervised Learning (WSL) deals with missing, inaccurate, or inexact annotations. Our problem is a particular case of learning with missing labels since each task-specific dataset provides a set of labels where the two sets are disjoint. Li and Hoiem (2017) proposed a method to learn a new task from a model trained on another task. This method combines DA through transfer learning and MTL. In the end, two models are created: One for the first task and one for the second one. Kim et al. (2018) extended this approach by using a knowledge distillation loss in order to create a unique multi-task model. This aims to alternatively learn one task without forgetting the other one. The WSL problem was thus decomposed into an MTL problem with aforementioned limitations for our specific use case.

This work proposes a new framework to perform a joint segmentation while dealing with task-specific, domain-shifted and hetero-modal datasets.

## 3 Tissue and lesion segmentation learning from hetero-modal and task-specific datasets: Problem definition

In order to develop a joint model, we first propose a mathematical variational formulation of the problem and introduce a network architecture to leverage existing hetero-modal and task-specific datasets for tissue and lesion segmentation.

### 3.1 Formal problem statement

Let *x* = (*x* ^1^,., x^M^) ϵ *X* = ℝ^*N×M*^ be a vectorized multimodal image and *y* ϵ *Y* = {0*,., C*}^*N*^ its associated tissue and lesion segmentation map. *Ν, M* and *C* are respectively the number of voxels, modalities and classes. Note that images modalities are assumed to be co-registered and resampled in the same coordinate space containing *N* voxels. Our goal is to determine a predictive function, parametrised by the weights *θ ϵ* Θ, *h_θ_: X* ↦ *Y* that minimises the discrepancy between the ground truth label vector *y* and the prediction *h_θ_(x)*. Let *L* be a loss function that estimates this discrepancy. Following the formalism used by Bottou et al. (2018), given a probability distribution *D* over *(X, Y)* and random variables under this distribution, we want to find θ* such that:

(1) $$\theta^{*}=\text{arg}{\text{min}}_{\theta }{Ε}_{\left(x,y\right)}{∼}_{D}\left[L\left(h_{\theta }\left(x\right),y\right)\right]$$

As is the norm in data-driven learning, we do not have access to the true joint probability *D*. In supervised learning, the common method is to estimate the expected risk using training samples. Given a set of *n* ε ℕ independently drawn multimodal scans with their associated tissue and lesion segmentation map ${\left\{\left(x_{k},y_{k}\right)\right\}}_{k=1}^{n},$ we want to find θ* that minimises the empirical risk:

(2) $$\theta^{*}=\text{arg}{\text{min}}_{\theta }\sum_{\text{k=1}}^{\text{n}}L\left(h_{\theta }\left(x_{k}\right),y_{k}\right)$$

However, in our multitask scenario, we cannot directly estimate the empirical risk since we do not have access to a fully annotated dataset for the joint task. Instead, we propose to leverage task-specific and hetero-modal datasets.

### 3.2 Task-specific and hetero-modal datasets

Let us assume that we have access to two datasets with either the tissue annotations *y^T^* or the lesion annotations *y^L^* (task-specificity). Let

$$\begin{array}{l} S_{control}={\left\{\left(\left(x_{k}^{1},.,x_{k}^{M_{T}}\right),y_{k}^{T}\right)\right\}}_{k=1}^{n_{T}}\\ S_{lesion}={\left\{\left(\left(x_{k}^{1},...,x_{k}^{M_{L}}\right),y_{k}^{L}\right)\right\}}_{k=1}^{n_{L}} \end{array}$$

denote these two training sets, where *M_T_, M_L_, n_T_* and *n_L_* are respectively the number of modalities in the *control* and the *lesion* sets and the size of these sets. Note that although we use the term *control* for convenience, we may expect to observe pathology with “diffuse” anatomical impact, e.g. from dementia. In addition, for the clarity of presentation, we highlight that the considered *lesions* in this work are either White Matter Hyperintensities (WMH) or gliomas.

Since such datasets are typically developed in the scope of either tissue or lesion segmentation (but not both), the set of observed modalities may vary from one dataset to another (hetero-modality). Importantly, in this work, we consider that only T_1_ scans are provided in the *control* dataset, while the *lesion* set contains either 1) the T_1_ and the FLAIR scans for WMH segmentation, or 2) the T_1_, contrast-enhanced T_1_ (T_1_c), *T*_2_ and FLAIR scans for glioma segmentation. The full set of modalities is consequently given by the modalities in the *lesion* set, while the *control* dataset will have missing modalities. In our specific use cases, the T_1_ modality is a shared modality across the different datasets. It will nonetheless be apparent that our method can be trivially adapted for other shared modalities.

### 3.3 On the distribution D in the context of heterogeneous databases

As we expect different distributions across heterogeneous databases, two probability distributions of (*X Y*) over *(X, Y)* can be distinguished:

- under *D_control_*, (*X Y*) corresponds to a multimodal scan and joint segmentation map of a patient without lesions (*Y* effectively being a tissue segmentation map).
- under *D_lesion_*, (*X Y*) corresponds to a multimodal scan and joint lesion and tissue segmentation map of a patient with lesions.

Since traditional tissue segmentation methods are not adapted in the presence of lesions, the most important and challenging distribution *D* to address is the one for patients with lesions, *D_lesion_*. In the remainder of this work, we thus assume that:

(H_1_) $$D≜D_{lesion}$$

### 3.4 Hetero-modal network architecture

**Fig. 2:**
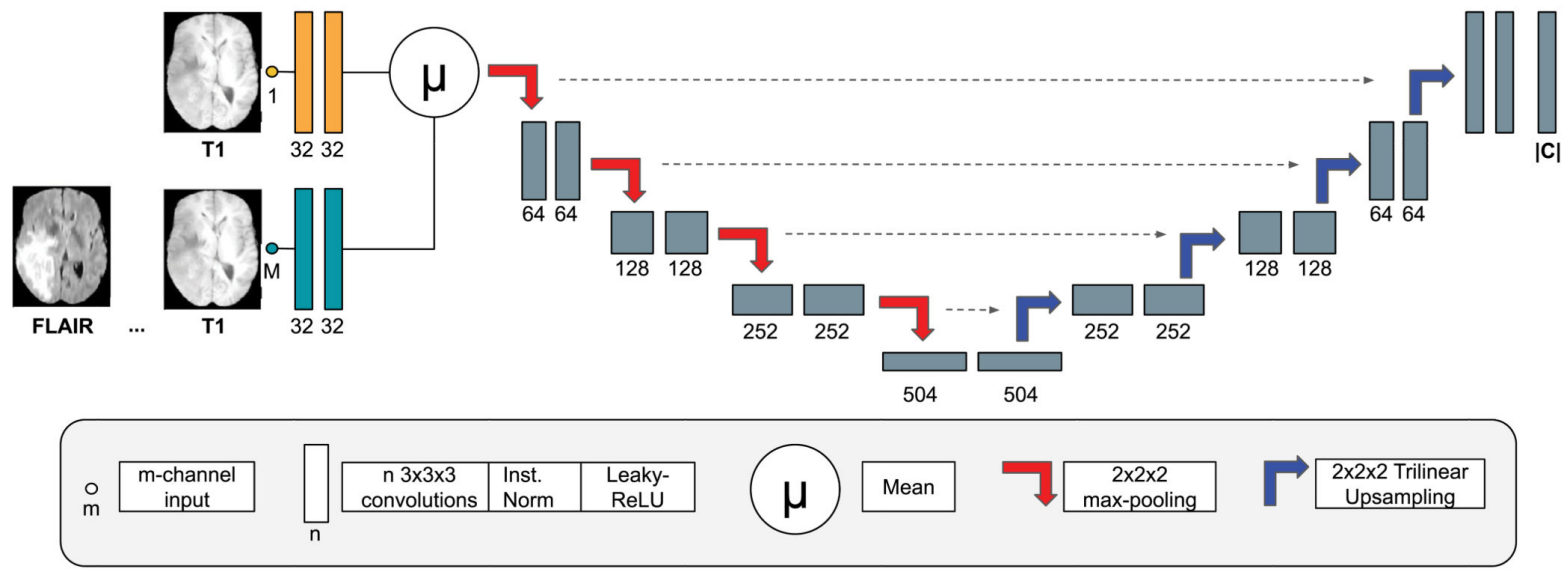
The proposed fully-convolutional network architecture: A mix a 3D U-Net (Çiçek et al., 2016a) and HeMIS (Havaei et al., 2016).

In order to learn from hetero-modal datasets, we need a network architecture that allows for missing modalities. Specifically, the input modalities are either a T_1_ scan or a full set of modalities. To deal with missing modalities, arithmetic operations are employed, as originally proposed in HeMIS (Havaei et al., 2016). The network architecture is based on a U-Net (Çiçek et al., 2016b), as shown in Fig. 2. Note that, while the proposed method requires a hetero-modal network, any specific architecture can be used. The proposed network is composed of two input branches, one for the T_1_ scan and one for the full set of modalities. Although HeMIS originally proposed to encode each modality independently, i.e one branch per modality, we experimentally found higher performance with these two branches. In the presence of the full set of modalities, features extracted from the T_1_ scan and all the modalities are averaged. Consequently, the network allows for missing modalities, i.e. is hetero-modal. This hetero-modal network with weights *θ* is used to capture the predictive function *h_θ_* that can accept either T_1_ or the full set of modalities as input.

## 4 Optimising tissue and lesion segmentation as a joint task

Given the mathematical formulation of the problem and the hetero-modal network architecture, we propose a method to empirically optimise the joint problem of tissue and lesion segmentation.

### 4.1 Loss decomposition

Let *C_T_, C_L_* and 0 be respectively the set of tissue classes and lesion classes and the value of the background class in the segmentation masks. Since *C_T_* and *C_L_* are disjoint, the segmentation map *y* can be decomposed into two segmentation maps *y =y^L^ +y^T^* with $y^{T}\in C_{T}\cup \left\{0\right\},y^{L}\in C_{L}\cup \left\{0\right\}$.

Let us assume that the loss function *L* can also be decomposed into a tissue loss function *L^T^* and a lesion loss function *L^L^*. This is common for multi-class segmentation loss functions in particular for those with *one-versus-all* strategies (e.g. Dice loss, Jaccard loss, Generalized Cross-Entropy). Then, the joint and multi-class segmentation problem can be formulated as a multi-task problem:

(H_2_) $$L\left(h_{\theta }\left(x\right),y\right)=L^{T}\left(h_{\theta }\left(x\right),y^{T}\right)+L^{L}\left(h_{\theta }\left(x\right),y^{L}\right)$$

In combination with (H_1_), (1) can be rewrittern as:

(3) $$\theta^{*}=\text{arg}{\text{min}}_{\theta }\underset{R^{T}}{\underset{︸}{{\text{E}}_{D_{lesion}}\left[L^{T}\left(h_{\theta }\left(x\right),y^{T}\right)\right]+}}\underset{R^{T}}{\underset{︸}{{\text{E}}_{D_{lesion}}\left[L^{L}\left(h_{\theta }\left(x\right),y^{L}\right)\right]}}$$

While the second expected risk *R^L^* can be estimated using the full set of modalities and the lesion annotations provided in the *lesion* dataset, the first expected risk *R^T^* appears to be intractable due to the missing tissue annotations in the *lesion* dataset. In the next sections, we first propose an upper bound of the expected tissue risk *R^T^* and then a means to estimate this upper bound using the *control* dataset.

### 4.2 Upper bound of the expected tissue risk R^T^

Although, thanks to its hetero-modal architecture, *h_θ_* may handle inputs with varying number of modalities, the current decomposition (3) assumes that all the modalities of *x* are available for evaluating the loss. In our scenario, the *control* set of scans with tissue annotations only contains the T_1_ scans. Consequently, as we do not have all the modalities with tissue annotations, and as naively evaluating a loss with missing modalities would lead to a bias, estimating *R^T^* is not straightforward.

Let us assume that the tissue loss function *L^T^* satisfies the triangle inequality:

(H_3_) $$\forall \left(a,b,c\right)\in y^{3}:L^{T}\left(a,c\right)\leq L^{T}\left(a,b\right)+L^{T}\left(b,c\right)$$

Although not all losses satisfy (H_3_), it is known that the binary Jaccard is a distance (Späth, 1981; Kosub, 2018) and thus satisfies the triangle inequality.

**Definition 4.1.** (Binary Jaccard distance)

The binary Jaccard distance *J_bin_* is defined such that:

(4) $$\forall a,b\in {\left\{0,1\right\}}^{N},J_{bin}\left(a,b\right)=1-\frac{\sum_{i=1}^{N}a_{i}b_{i}}{\sum_{i=1}^{N}a_{i}+b_{i}-a_{i}b_{i}}$$

However, network outputs are typically pseudo-probabilities, and the soft version of (4) does not satisfy the triangle inequality. To satisfy (H_3_), we extend the binary Jaccard distance to a multi-class probabilistic formulation that coincides with the binary Jaccard for binary inputs but preserves the metric property for probabilistic inputs.

**Definition 4.2.** (Probabilistic multi-class Jaccard distance)

Let *C* be the number of classes in *C, N* be the number of voxels and $P\subset {\left[0,1\right]}^{C\times N}$ denote the set of probability vector maps such that for any $p={\left(p_{c,i}\right)}_{c\in C,i\in \left[0;N\right]}\in P$:

$$\forall i\in \left[0;N\right],\sum_{c\in C}p_{c,i}=1$$

The probabilistic multi-class Jaccard distance is defined for any *(u, v)* ∈ *P^2^* as:

(5) $$J\left(u,v\right)=\sum_{c\in C}\omega_{c}\frac{2\sum_{i=1}^{N}\left|u_{c,i}-v_{c,i}\right|}{\sum_{i=1}^{N}\left|u_{c,i}\right|+\left|v_{c,i}\right|+\left|u_{c,i}-v_{c,i}\right|}$$

where *ω_c_* are the class weights such that with $\sum_{c\in C}\omega_{C}=1$

As shown in A.1, the binary and probabilistic Jaccard distance coincide on the set of binary vectors {0, 1}^N^. Furthermore, (5) satisfies (H_3_).

**Lemma 4.1.** *The probabilistic multi-class Jaccard distance is a distance and thus satisfies the triangle inequality*.

**Proof.** The proof, detailed in A.2, follows from the Steinhaus transform (Späth, 1981) applied to the metric space ([0, 1]^N^, *d1)* where *d1* is the distance induced by the *L1* norm. D

Under (H_3_), *L^T^* satisfies the following inequality:

(6) $$L^{T}\left(h_{\theta }\left(x\right),y^{T}\right)\leq L^{T}\left(h_{\theta }\left(x\right),h_{\theta }\left(x^{T_{1}}\right)\right)+L^{T}\left(h_{\theta }\left(x^{T_{1}}\right),y^{T}\right)$$

where *x^T^* ^1^ denotes the T_1_ scan associated to *x*. Consequently, we find an upper bound of the expected tissue risk:

(7) $${ℜ}^{T}\left(\theta \right)\leq \underset{{ℜ}_{{\text{T}}_{\text{1}}\to \text{Full}}^{\text{T}}}{\underset{︸}{{\text{E}}_{D_{lesion}}\left[L^{T}\left(h_{\theta }\left(x\right),h_{\theta }\left(x^{T_{1}}\right)\right)\right]+}}\underset{{ℜ}_{{\text{T}}_{\text{1}}}^{\text{T}}}{\underset{︸}{{\text{E}}_{D_{lesion}}\left[L^{T}\left(h_{\theta }\left(x^{T_{1}}\right),y^{T}\right)\right]}}$$

Minimising ${ℜ}_{{\text{T}}_{\text{1}}}^{\text{T}}$ enforces the network to generate accurate tissue segmentation using only T_1_ as input. Minimising ${ℜ}_{{\text{T}}_{\text{1}}\to Full}^{\text{T}}$ encourages consistency between the outputs when given only T_1_ or the full set of modalities as input. This latter term allows for transferring the knowledge learnt on the T_1_ scan to the full set of modalities.

An empirical estimation of *R^T^_T__F__ull_* can be obtained by comparing the network outputs using either T_1_ or the full set of modalities as input. In contrast, *R^T^_T_* requires comparison of inference done, under *D_lesion_*, from T_1_ inputs with ground truth tissue maps *y^T^*. While this provides a step towards a practical evaluation of *R^T^*, we still face the challenge of not having tissue annotations *y^T^* under *D lesion*.

### 4.3 Estimating ${ℜ}_{{\text{T}}_{\text{1}}}^{\text{T}}$ using the control *dataset*

To estimate ${ℜ}_{{\text{T}}_{\text{1}}}^{\text{T}}$, we assume that the neural network *h_θ_* is invariant to a potential domain shift between the T_1_ scans of the *control* and *lesion* datasets on the non-lesion regions. Specifically, we assume that the restriction of the feature distributions (rather than the image intensity distributions) over *D_lesion_* and *D_control_* to the non-lesion parts of the brain (i.e. the voxels *i* such that *y_i_* ∈ *C_T_)* are comparable, i.e.:

(H_4_) $${\text{P}}_{D_{lesion}}\left(h_{\theta }{\left(x^{T_{1}}\right)}_{i},y_{i}|y_{i}\in C_{T}\right)={\text{P}}_{D_{control}}\left(h_{\theta }{\left(x^{T_{1}}\right)}_{i},y_{i}|y_{i}\in C_{T}\right)$$

This means that the neural network *h_θ_* generates similar outputs on the non-lesion parts of the brain between the two datasets, leading to:

(8) $${ℜ}_{{\text{T}}_{\text{1}}}^{\text{T}}={\text{E}}_{D_{lesion}}\left[L^{T}\left(h_{\theta }\left(x^{T_{1}}\right),y^{T}\right)\right]={\text{E}}_{D_{control}}\left[L^{T}\left(h_{\theta }\left(x^{T_{1}}\right),y^{T}\right)\right]$$

Consequently, under (H_4_), *R^T^_T_* can be estimated using the *T*_1_ scans and their tissue annotations in the *control* dataset. Section 5 presents means of ensuring that assumption (H_4_) is satisfied even in the presence of domain shift in the image intensity distributions.

### 4.4 Summary of the expected risk decomposition

Bringing all the terms together, given (3), (7) and (8), we seek the parameters *θ ∗* that optimise the tractable upper bound *R_s_eg* of the (intractable) expected risk:

(9) $$\theta^{*}=\text{arg}{\text{min}}_{\theta }\left\{{ℜ}_{seg}={\text{E}}_{D_{control}}\left[L^{T}\left(h_{\theta }\left(x^{T_{1}}\right),y^{T}\right)\right]+{\text{E}}_{D_{lesion}}\left[L^{L}\left(h_{\theta }\left(x\right),y^{L}\right)+L^{T}\left(h_{\theta }\left(x\right),h_{\theta }\left(x^{T_{1}}\right)\right)\right]\right\}$$

## 5 Matching feature distributions across datasets

In this section, we explore different approaches that ensure the feature distributions extracted from the *control* and *lesion* T_1_ scans are comparable, i.e. we want to satisfy (H_4_) even in the presence of domain shift.

### 5.1 *Similar acquisition protocols for the* control *and* lesion *datasets*

Let’s first assume that the acquisition protocols are similar for the *control* and *lesion* datasets, i.e. they are not domain-shifted. Specifically, we assume that the T_1_ images have been acquired with similar sequences, spacial resolution and field strength. In this case, similar to Chen and Konukoglu (2018), the restriction of the distributions *D_lesion_* and *D_control_* to the non-lesion parts of the brain can be assumed to be the same on the T_1_ scans, i.e.:

(10) $${\text{P}}_{D_{lesion}}\left(\chi_{i}^{T_{1}},y_{i}|y_{i}\in C_{T}\right)={\text{P}}_{D_{control}}\left(\chi_{i}^{T_{1}},y_{i}|y_{i}\in C_{T}\right)$$

In the absence of domain shift, we can reasonably assume that the network produces similar outputs on the non-lesion parts of the brain for the two distributions, i.e. that (H_4_) is satisfied. No specific additional action thus needs to be implemented.

### 5.2 Generating pseudo-healthy scans to learn tissue segmentation from domain-shifted *T*_1_ scans

Let’s now consider the presence of a domain shift between the *T_1_ control* and *lesion* scans due to different acquisition protocols. In this section, we propose to synthesise pseudo-healthy scans from domain-shifted T_1_ lesion scans in order to extend the *control* dataset with *control* scans associated to the protocol of acquisition of the *lesion* dataset. Since the *control* and *lesion* datasets are domain-shifted, existing lesion removal approaches, based either on CycleGANs (Xia et al., 2019; Sun et al., 2018) or Variational Autoencoders (Chen and Konukoglu, 2018), are not adapted as they require training data with no domain shift beyond the presence of absence of pathology.

To tackle the presence of an acquisition-related domain shift, we propose to generate pseudo-healthy scans and their annotations using traditional image computing techniques that are inherently robust to different acquisition protocols. For example, for white matter lesions, lesion filling methods allow for transforming scans with lesions into pseudo-healthy scans (Valverde et al., 2014; Prados et al., 2016). For large and unilateral pathology, we propose to synthesise pseudo-healthy T_1_ scans by symmetrising the “healthy” hemisphere of the patients with lesions located in one hemisphere only. The inter-hemispheric symmetry plane is estimated via the technique described in Prima et al. (2002). Finally, the “healthy” hemisphere of those patients is mirrored in order to create a symmetric pseudo-healthy brain. Having generated pseudo-healthy images, traditional methods, designed for *control* scans, such as the GIF framework (Cardoso et al., 2015), can then be employed to generate the corresponding bronze standard tissue annotations.

With this set of scans $S_{pseudo}^{T_{1}}$, we have access to a *pseudo-control* dataset acquired with a similar protocol as in the *lesion* dataset and similar on the non-lesion part of the brain, and thus are in the scenario described in 5.1. Let denote $D_{pseudo}^{T_{1}}$ the distribution of those scans. The expected tissue risk ${ℜ}_{{\text{T}}_{\text{1}}}^{\text{T}}$ is then equal to the expect tissue risk under $D_{pseudo}^{T_{1}}$:

(11) $${ℜ}_{{\text{T}}_{\text{1}}}^{\text{T}}={\text{E}}_{D_{lesion}}\left[L^{T}\left(h_{\theta }\left(x^{T_{1}}\right),y^{T}\right)\right]={\text{E}}_{D_{pseudo}^{T_{1}}}\left[L^{T}\left(h_{\theta }\left(x^{T_{1}}\right),y^{T}\right)\right]$$

To take advantage of the manual annotations in the *control* dataset, we resort to averaging the two formulations (8,11):

(12) $${ℜ}_{{\text{T}}_{\text{1}}}^{\text{T}}\approx \frac{{\text{E}}_{D_{control}}\left[L^{T}\left(h_{\theta }\left(x^{T_{1}}\right),y^{T}\right)\right]+{\text{E}}_{D_{pseudo}^{T_{1}}}\left[L^{T}\left(h_{\theta }\left(x^{T_{1}}\right),y^{T}\right)\right]}{2}$$

Consequently, the expected tissue risk *R^T^_T_* can be estimated using the *control* and *pseudo-control *T*_1_* scans.

### 5.3 Alternative unsupervised DA techniques

In order to satisfy (H_4_), the feature representation of the non-lesion parts of the brain has to be invariant to the changes induced by the different protocols. A direct way to align the feature distributions restricted to the non-lesion parts would be to match the representations of pairs of scans acquired with the different settings. However, in our scenario, we do not have access to such pairs of domain-shifted scans.

In contrast, unsupervised DA allows to perform domain adaptation using unpaired and non-annotated domain-shifted scans. Unsupervised DA techniques commonly introduce an additional term *(R_DA_)* that encourages the network to be invariant to the domain shift. Then, the total expected risk reads:

(13) $${ℜ}_{total}={ℜ}_{seg}+\lambda {ℜ}_{DA}$$

where *λ* is a hyper-parameter that allows for balancing the segmentation risk *R_seg_* ((9)) with the DA regularisation *R_DA_*.

The definition of the DA term depends on the DA technique. In this work, two common unsupervised DA methods are considered, based either on data augmentation or adversarial learning.

#### 5.3.1 Unsupervised DA via physically-inspired data augmentation

Since T_1_ scans play a key role for structure analysis, we expect high-resolution T_1_ scans for datasets developed in the scope of tissue segmentation, such as the *control* dataset. Conversely, T_1_ scans are often less critical for lesion segmentation and T_1_ scans may have been acquired with a lower resolution.

Let’s assume that, less effort has been done to acquire high-resolution *T_1_ lesion* scans, explaining the differences in acquisition protocols. Specifically, we assume that the domain shift is caused by the presence of T_1_ *lesion* scans with artefacts (e.g. related to the MR bias field or the presence of motion artefacts) and a lower acquisition resolution. We additionally assume that differences of scanner characteristics (manufacturer, field strength) are excluded.

Then, physically-informed augmentation such as random bias field (Sudre et al., 2017) and motion artefacts (Shaw et al., 2019) and spacial smoothing can be employed to generate scans that are similar to the *T_1_ lesion* scans. Let denote *Tψ* the composition of these transformations parametrised by the parameters *ψ* ∼ *Dψ*. For any T_1_ *control* scan $x_{C}^{T_{1}}$, we can thus generate an augmented version $T_{\phi }\left(x_{C}^{T_{1}}\right)$, i.e. getting access to pairs of domain-shifted T_1_ scans. This allows to minimise the discrepancy between the feature representations learnt by the neural network across the two domains by enforcing consistency across outputs from paired domain-shifted inputs, i.e.:

(14) $${ℜ}_{DA}={\text{E}}_{D_{\psi }}{\text{E}}_{D_{control}}\left[L^{T}\left(h_{\theta }\left(x_{c}^{T_{1}}\right),h_{\theta }\circ T_{\phi }\left(x_{c}^{T_{1}}\right)\right)\right]$$

An empirical estimation of the DA regularisation term *R_DA_* is obtained by comparing the network outputs using the T_1_ *control* scans and their augmented versions as input.

Consequently, if the domain shift is due to different spatial resolutions and the presence of the aforementioned artefacts, the network can be trained to be invariant to the domain shift, i.e. to satisfy (H_4_).

#### 5.3.2 Unsupervised DA via adversarial learning

Let’s now assume that the domain shift cannot easily be simulated. In this case, we can use adversarial learning. Adversarial approaches for domain adaptation can be seen as a two-player game: A discriminator *Dφ*, parametrised by the weights *φ ∈* Φ, is trained to distinguish the source domain features from the target domain features, while the segmentation network *h_θ_* is simultaneously trained to confuse the domain discriminator.

The discriminator aims to predict the probability that extracted features are part of the *lesion* feature distributions. The discriminator accuracy can thus be seen as a measurement of the discrepancy between the *lesion* and *control* feature distributions and used as a DA regularisation term:

(15) $${ℜ}_{DA}\left(\phi ,\theta \right)={\text{E}}_{D_{lesion}}\left[1-D_{\phi }\left(h_{\theta }\left(x\right)\right)\right]+{\text{E}}_{D_{control}}\left[D_{\phi }\left(h_{\theta }\left(x\right)\right)\right]$$

This DA term can be estimated by using features extracted from *T*_1_ *control* and *lesion* scans as input of the discriminator.

The following proposition shows that the discriminator accuracy is a principled measurement of the feature distribution discrepancy:

**Proposition 1.** *Let assume that L satisfies the triangle inequality and is bounded. Let us also assume that the family of domain discriminators* $H_{\Phi }={\left\{D_{\phi }\right\}}_{\phi \in \Phi }$ *is rich enough. Then there is a constant K such that*:

(16) $${\text{E}}_{D_{lesion}}\left[L\left(h_{\theta }\left(x\right),y\right)\right]\leq {ℜ}_{seg}+K\underset{\phi }{\sup}{ℜ}_{DA}\left(\phi ,\theta \right)+\in \left(\Theta \right)$$

*where* ε(Θ) *is independent of the network parameters θ and corresponds to the accuracy of the best (and unknown) segmenter in the family of functions parametrised in* Θ.

**Proof.** *The proof uses (3), (7), is based on Ben-David et al. (2010) and Long et al. (2018) and detailed in Appendix B*. D

(16) shows that the intractable expected loss is bounded by a weighted sum of the tractable segmentation risk (9) and the accuracy of the best discriminator, up to a constant w.r.t the network parameters.

Moreover, the alternative optimisation strategy can be seen as a way to estimate the best discriminator while minimising the upper bound defined in (16).

Note that (16) stands for features extracted at any level of *h_θ_*. In this work and similarly to Orbes-Arteaga et al. (2019), the contracting path features from the U-Net are used as input of the discriminator.

## 6 Implementation of the joint model optimisation

Given the formulation of the joint model and our proposed computationally tractable decomposition, we present in this section the implementation of our framework.

### 6.1 Stochastic optimisation of the joint model

**Fig. 3:**
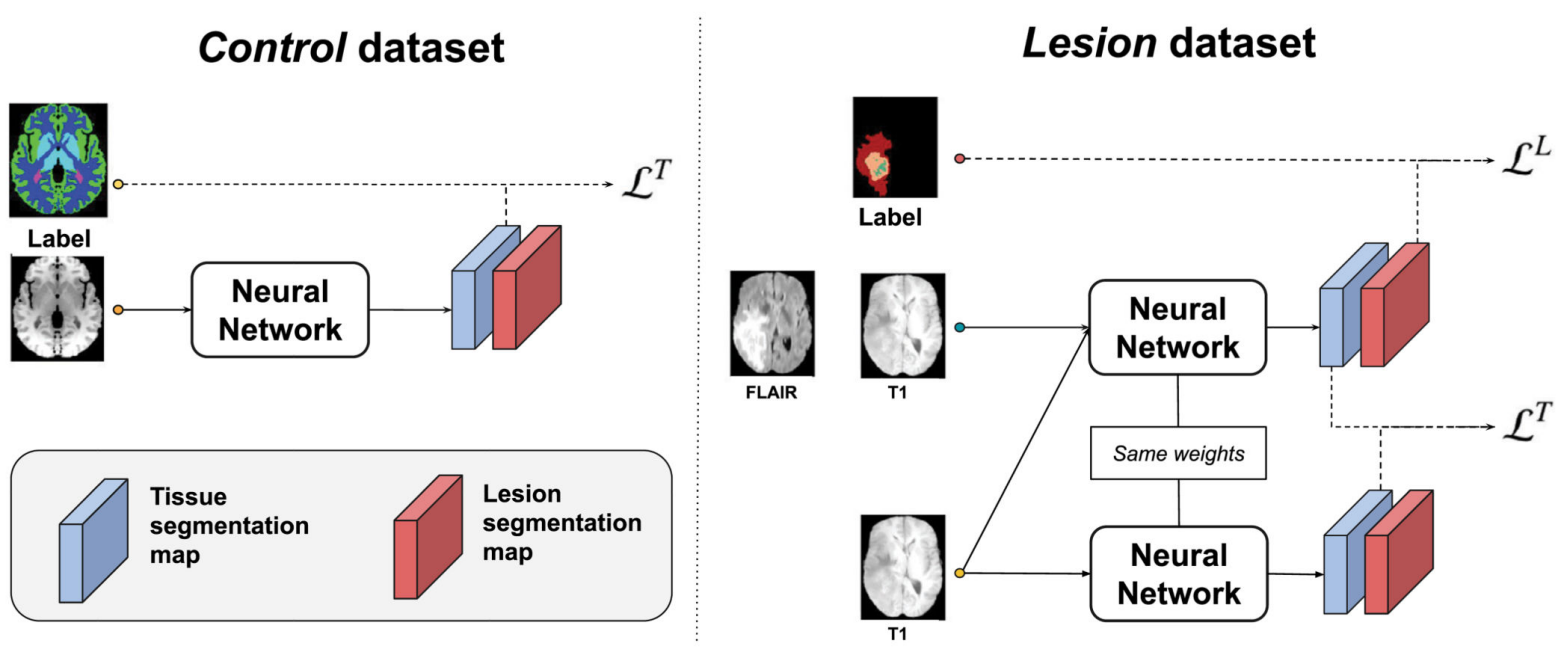
The training procedure using samples from the *control* and *lesion* datasets. The different elements of the decomposed loss upper bound are computed and minimised at each training iteration. The same network is used for all the different hetero-modal inputs. Note that domain adaptation is not represented.

We use a stochastic gradient descent approach to minimise the expected risk decomposition (9) and to enforce the network to be invariant to a potential domain shift between the datasets. The total loss function reads:

(17) $$L_{total}=L_{seg}+\lambda L_{\text{DA}}$$

where *λ* is a hyper-parameter that allows for balancing the segmentation loss *Lseg* (associated to *R_s_eg)* with the domain adaptation loss *L_DA_* (associated to *R_DA_)*. Fig. 3 shows the training procedure without DA. The weights of the segmentation loss are given by the decomposition of the problem. The domain adaptation parameter *λ* is a hyper-parameter that is experimentally chosen.

At each training iteration, we draw pairs of samples $\left(x_{l},y_{l}^{L}\right)$ and $\left(x_{C}^{T_{1}},y_{C}^{T}\right)$ from *S_lesion_* and *S_control_* and compute in each mini-batch the following loss functions and associated gradient. Note that there is no natural pairing between $\left(x_{l},y_{l}^{L}\right)$ and $\left(x_{C}^{T_{1}},y_{C}^{T}\right)$. Our paired sampling procedure thus exploits random pairing.

As presented in Section 5, different scenarios are considered.

**Similar acquisition protocols** If the datasets are not domain-shifted, no DA is required (*λ* = 0 *)*, and the segmentation loss is:

(18) $$\begin{array}{l} L_{seg}=L^{L}\left(h_{\theta }\left(x_{1}\right),y_{l}^{L}\right)+L^{L}\left(h_{\theta }\left(x_{1}\right),h_{\theta }\left(x_{l}^{T_{1}}\right)\right)\\ +L^{L}\left(h_{\theta }\left(x_{c}^{T_{1}}\right),y_{c}^{T}\right) \end{array}$$

We experimentally found that the inter-modality tissue loss ${ℜ}_{{\text{T}}_{\text{1}}\to Full}^{\text{T}}$ has to be skipped for few epochs (50 in our experiments).

**Pseudo-healthy generation** Given a pseudo-healthy annotated set of scans $S_{pseudo}^{T_{1}}=\left\{x_{pseudo}^{T_{1}},y_{pseudo}^{T}\right\}$, no DA is employed (*λ* = 0), and the segmentation loss, defined by (12), is:

(19) $$\begin{array}{l} L_{seg}=L^{L}\left(h_{\theta }\left(x_{1}\right),y_{l}^{L}\right)+L^{L}\left(h_{\theta }\left(x_{1}\right),h_{\theta }\left(x_{l}^{T_{1}}\right)\right)\\ +\frac{1}{2}\left[L^{T}\left(h_{\theta }\left(x_{pseudo}^{T_{1}}\right),y_{pseudo}^{T}\right)+L^{L}\left(h_{\theta }\left(x_{c}^{T_{1}}\right),y_{c}^{T}\right)\right] \end{array}$$

**DA via augmentation** If we assume that the differences of protocols can be simulated (random bias field, motion artefacts and spatial smoothing), the domain invariance (14) is learnt by minimising the inter-domain feature discrepancy defined as:

$$L_{DA}=L^{T}\left(h_{\theta }\left(x_{c}^{T_{1}}\right),h_{\theta }\left(T_{\phi }\left(x_{c}^{T_{1}}\right)\right)\right)$$

where *Tφ* corresponds to a composition of theses transformations. The segmentation loss *L_s_eg* is the same as in (18).

**DA via adversarial learning** If adversarial learning is employed, a discriminator *D* is trained to discriminate scans from the two domains by maximising the domain classification accuracy. For computational stability, the *L1* distance defined in (15) has been replaced by the cross-entropy. Conversely, the segmenter *h_θ_* is train to minimise this domain classification accuracy, i.e.:

$$L_{DA}=\text{log}\left(D_{\psi }\left(h_{\theta }\left(x_{c}^{T_{1}}\right)\right)\right)+\text{log}\left(1-D_{\psi }\left(h_{\theta }\left(x_{l}^{T_{1}}\right)\right)\right)$$

As in Kamnitsas et al. (2017); Orbes-Arteaga et al. (2019), the DA loss is skipped for few epochs (20 in our experiments) in order to initialise the discriminator. The segmentation loss *L_s_eg* is the same as in (18).

### 6.2 Implementation details

We implemented our network in PyTorch, using TorchIO (Pérez-García et al., 2020). Codes are available at http://github.com/ReubenDo/jSTABL.

Convolutional layers are initialised such as proposed in He et al. (2015). The scaling and shifting parameters in the batch normalisation layers were initialised to 1 and 0 respectively. As suggested by Ulyanov et al. (2016), we used instance normalisation. We used the same discriminator as in Orbes-Arteaga et al. (2019).

We performed a 3-fold cross validation. For each fold, we randomly split the data into 70% for training, 10% for validation and 20% for testing. We used a batch of 2 *lesion* scans, and 2 *control* scans. Note that, for the DA approach based on data augmentation, the batch of 2 *control* scans consists in a pair of non-augmented/augmented *control* scans. As a data augmentation, a rotation with a random angle in [-10◦, 10◦] and a random Gaussian noise are employed. The network was trained using Adam optimiser (Kingma and Ba, 2015) the learning rates *l_R_, β1, β_2_* were initially respectively set up to 5.10^-4^, 0.9 and 0.999. *l_R_* was progressively reduced by a factor of 2 every 10,000 iterations. We employed the training strategy used for the nnU-Net (Isensee et al., 2019): The learning rate is reduced by a factor 2 after 15 epochs without reduction of the exponential moving average of the loss on the validation split.

We used the probabilistic version of the multi-class Jaccard distance (5) as the segmentation loss function. In order to give the same weight to the lesion segmentation and the tissue segmentation, we choose *ω* such that

$$\sum_{c\in C_{tissue}}\omega_{c}=\sum_{c'\in C_{lesion}}\omega_{c'}=\frac{1}{2}$$

## 7 Experiments and results

### 7.1 Joint white matter lesion and tissue segmentation

#### 7.1.1 Task and datasets

In this first set of experiments, we focus on the segmentation of white matter lesions and six tissue classes (white matter, grey matter, basal ganglia, ventricles, cerebellum, brainstem), as well as the background. As detailed in Table 1, we used 2 *control* datasets and 2 *lesion* datasets:

- **Lesion data S_lesion_**: The White Matter Hyperintensities (WMH) training database (Kuijf et al., 2019) consists of 60 sets of brain MR images (T_1_ and FLAIR, *M* = 2) with manual annotations of WMHs. The data comes from three different institutes. Note that images modalities are be co-registered and resampled in the FLAIR coordinate space.
- **Tissue data S_control_**: Consists of 35 T_1_ scans (*M*’ = 1 *)* from the OASIS project (Marcus et al., 2007) with annotations of 143 structures of the brain provided by Neuromorphometrics, Inc. (http://Neuromorphometrics.com/) under academic subscription. From the 143 structures, we deduct the 6 tissue classes. In order to have balanced training datasets between the two datasets, to include data acquired at the same field strength (3T) as the *lesion* data, and similar to Li et al. (2017), we added 25 T_1_ control scans from the Alzheimer’s Disease Neuroimaging Initiative 2 (ADNI-2) database (Jack Jr. et al. (2008), adni.loni. usc.edu) with bronze standard parcellation of the brain structures computed with the accurate but time-consuming algorithm of Cardoso et al. (2015).
- **Fully annotated data S_fully_:** MRBrainS18 (http://mrbrains18.isi.uu.nl/) is composed of 30 sets of brain MR images with tissue and lesion manual annotations. 7 scans are publicly available for training and validation. Although the cerebrospinal fluid (CSF) has been annotated in MRBrainS18, it was considered as background to have the same set of tissue classes as in **S_contro_l** where the CSF was not labelled. Note that image modalities are be co-registered and resampled in the FLAIR coordinate space.

#### 7.1.2 Similar acquisition protocol for the T_1_ scans

Despite the differences in scanners, all T_1_ acquisitions across the datasets followed very similar protocols (MP-RAGE) (see Table 1). Therefore, they were considered as following a similar distribution and the data was only pre-processed as follows.

- **Skull stripping:** All the scans were skull-stripped using ROBEX (Iglesias et al., 2011).
- **Resampling:** All the scans in **S_control_** are resampled into the transversal direction with slices of 3 mm thickness to obtain a similar spacing 1×1×3 mm^3^ in the datasets.
- **Intensity normalisation:** We used a zero-mean unit-variance normalisation in order to match the intensity distributions.

#### 7.1.3 Description of the compared models

We considered three different models in our experiments.

- **Pipeline model (*Pipeline*):** This model corresponds to the com bination of two task-specific models:
  A *Tissue* segmentation model that only performs tissue segmentation and is trained on the *T*_1_ scans from the dataset with tissue annotations **S_control_**.
  A *Lesion* model that only performs lesion segmentation and is trained using the *T*_1_ and FLAIR scans from the dataset with lesion annotations **S_lesion_**.
  The two models are combined such that the predicted lesion mask has the priority over the predicted tissue mask. Consequently, the background of the *Lesion* output is replaced by the *Tissue* output.
- **Fully-supervised model (*Fully-Sup*):** This joint model performs tissue and lesion segmentation and is trained using the *T*_1_ and FLAIR scans from the small fully-annotated dataset **S_fully_**.
- **Proposed joint model (*jSTABL*):** Our proposed model for joint Segmentation of Tissues and Brain Lesions is trained using both the *T*_1_ scans from **S_control_** with the tissue annotations and the *T*_1_ and FLAIR scans from **S_lesion_** with the lesion annotations.

Each model used the architecture presented in Fig. 2. Consequently, the *Pipeline* model has twice as many parameters as the other models.

In this set of experiments, the skull-stripped images are first cropped to remove the blank spaces and then padded to size of (144,192,48).

#### 7.1.4 Method for assessing the models

The performance of the three models was evaluated on the three datasets using Dice Score and 95% Hausdorff distance. On the *control* data (OASIS1+ADNI2) and WMH, scores were computed on the testing splits, while on MRBrainS18, models were submitted to the challenge MRBrainS18.

For the *control* data (OASIS1+ADNI2) and MRBrainS18, the full set of annotations allows a direct assessment of the tissue and the lesion segmentation performance. For WMH, only the *lesion* annotations are provided. In order to assess both the tissue and lesion segmentation on WMH, the lesions are filled as normal-appearing white matter on *T*_1_ images using the method described in Prados et al. (2016) and implemented in NiftySeg (Cardoso et al., 2015). Then, GIF framework (Cardoso et al., 2015) was performed on the modified *T*_1_ scans to obtain bronze standard tissue annotations. The tissue mask and lesion annotations were then merged by completing the non-lesion parts with the tissue mask. Finally, the model outputs are compared to the merged tissue and lesion masks. In the end, for each model and each dataset, we can assess the performance of tissue and lesion segmentation.

Given that participants to the MRBrainS18 challenge do not have access to the held-out evaluation data set and that the Jac-card score is not provided by the challenge organisers, only the Dice Similarity Coefficient (DSC) and 95th-percentile Hausdorff distance are reported for each class.

#### 7.1.5 Results

The main results are shown in Table 2 for the Dice Similarly Coefficient and in Table 3 for the 95th-percentile Hausdorff distance.

Firstly, our proposed method (*jSTABL*) achieves comparable performance to the single-task models on the *control* data (*Tissue*) and on WMH (priority of *Lesion* in *Pipeline*). This suggests that learning from hetero-modal datasets via our method does not degrade the performance on the tasks characterising the task-specific datasets.

**Fig. 4:**
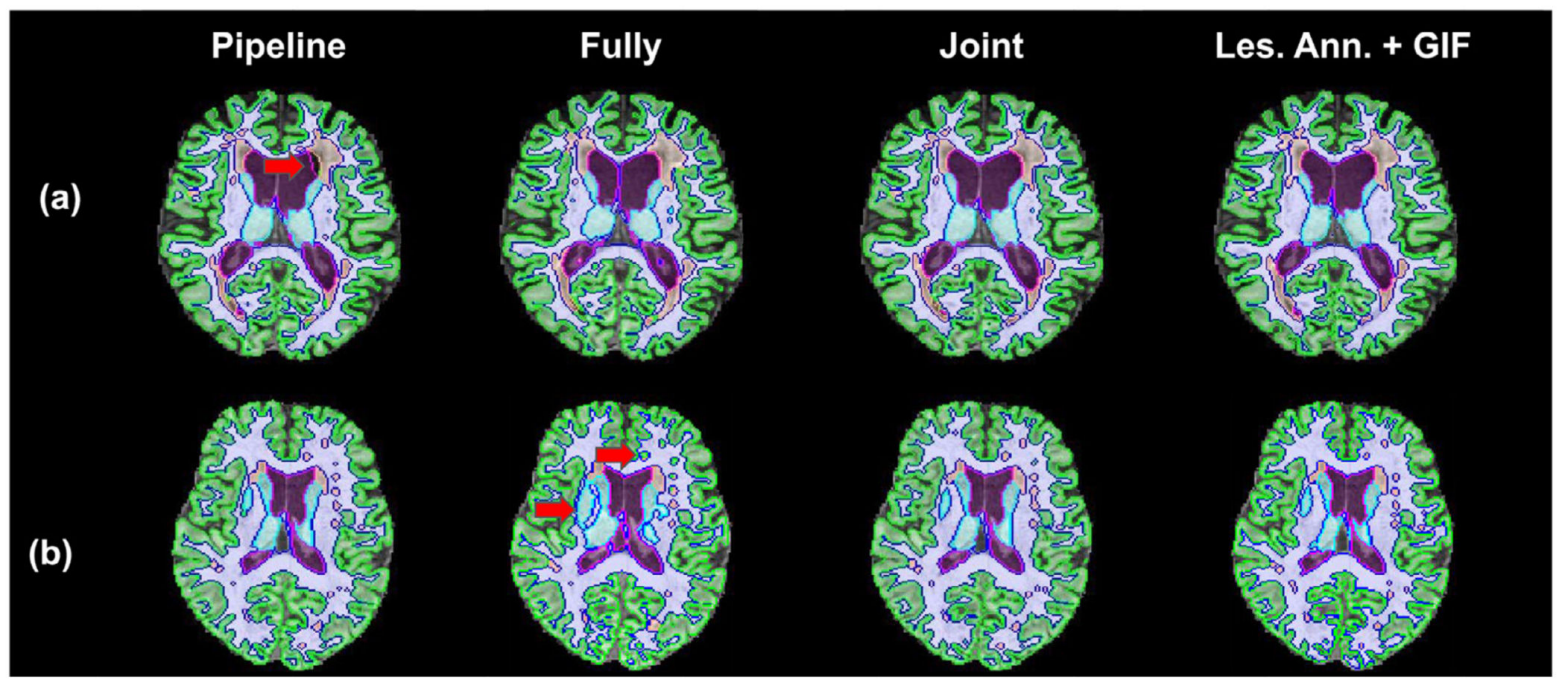
Examples of output for the different models: *Pipeline; Fully-sup; jSTABL* ; and the combination of the manual annotation and GIF. (a) *Tissue* model used in *Pipeline* can be perturbed by the presence of lesions (arrow). (b) Example for which the fully-supervised model largely fails to segment the tissue and the lesions.

Secondly, *jSTABL* slightly outperforms *Pipeline* on segmenting the tissues in WMH for the two sets of metrics. This shows that the tissue knowledge learnt from *T*_1_ scans has been well generalised to multi-modal scans. Although we could have expected that the presence of lesions would create perturbations for the *Tissue* model, this latter model in fact ignores the lesions and mostly classifies them as white matter. Given that the white matter lesions are usually surrounded by white matter, the *Pipeline* predictions are consequently not too degraded. However, some artefacts around the lesions in the *Pipeline* outputs can be observed, in particular in the ventricles for patients with large lesions surrounding them. Fig. 4(a) shows an example for which parts of the ventricles are classified as background. In contrast, we did not observe such artefacts with *jSTABL* predictions.

**Fig. 5:**
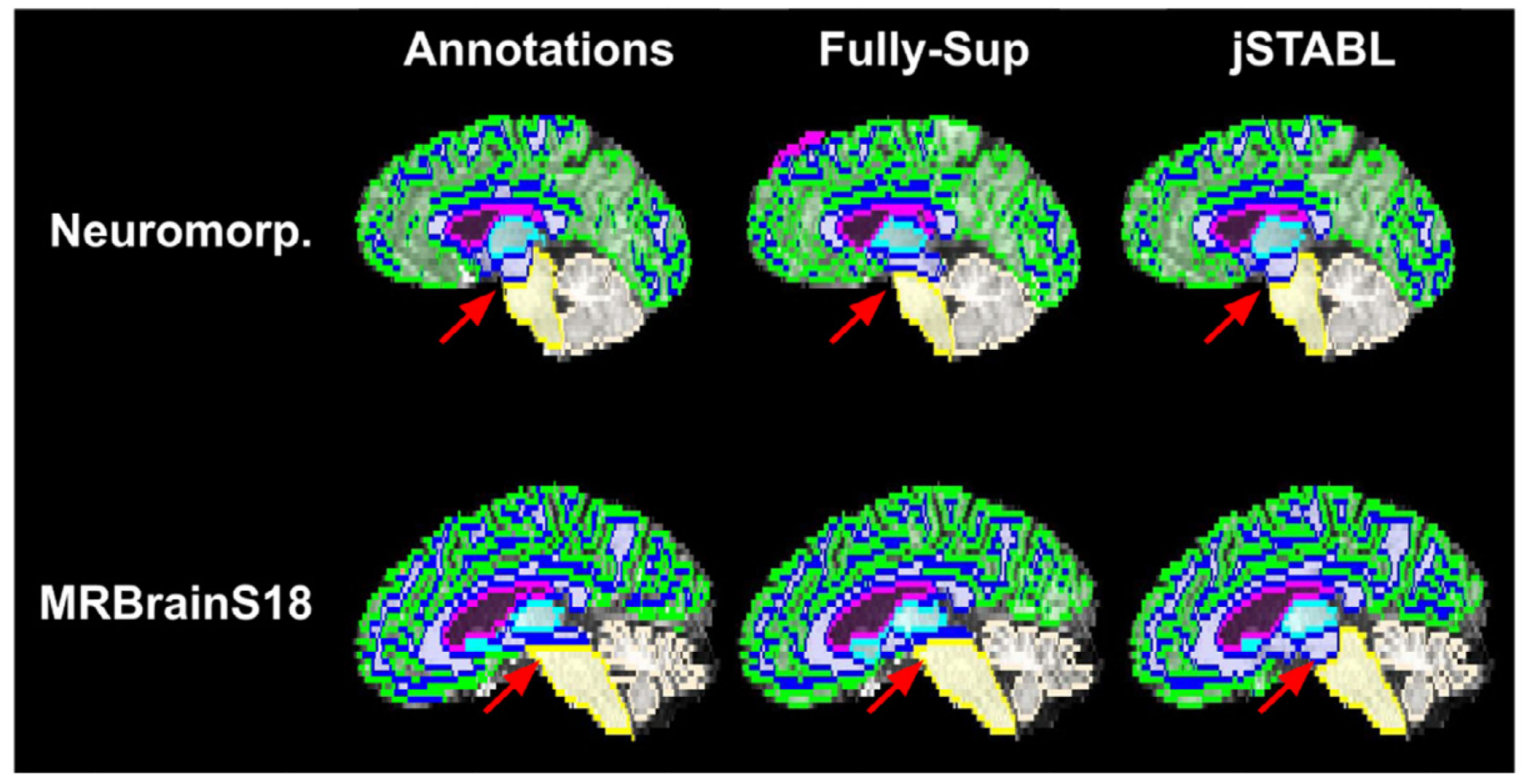
Comparison of the brainstem annotations by Neuromorphometrics and in MRBrainS18 and between the outputs of the *Fully-Sup* and *jSTABL* models. Arrows show the annotations protocol differences.

Thirdly, *jSTABL* outperforms the fully-supervised model (*Fully-Sup*) on the *control* data (OASIS1+ADNI2) and WMH, while reaching comparable performance on MRBrainS18. This demonstrates the two main advantages of our method. First, without using any fully-annotated data, our model performs as well as a fully-supervised model that could be considered as an upper bound for our method, especially when the testing and training splits are from the same dataset (MRBrainS18). Secondly, our method takes advantage of large task-specific datasets: Unlike the fully-supervised model (*Fully-Sup*), *jSTABL* generalises well on unseen data (MRBrainS18). While the fully-supervised model (*Fully-Sup*) fails to segment scans from OASIS1, ADNI2 and WMH, the *jSTABL* model obtains relatively good performance on all the datasets we use for tissue and lesion segmentation. In particular, *jSTABL* outperforms SPM on 6 of the 7 classes. In fact, the only class that is significantly underperformed compared to the fully-supervised model (*Fully-Sup*) is the brain stem. This is due to observed differences in the annotation protocol across the *control* and MRBrainS datasets. Fig. 5 shows these differences and the consequences on the prediction.

### 7.2 Glioma and tissue segmentation

#### 7.2.1 Task and datasets

Additionally, we assess our framework on another main types of brain lesions: Gliomas. Our goal is to segment the 6 tissue classes and three tumour classes (whole tumour, core tumour, enhancing tumour). In this case, domain adaptation was required and its evaluation is the focus of this section. We used two sets of data in these experiments.

- **Tissue data S_control_:** again we used OASIS1 data and the same 25 *T*_1_ *control* scans from ADNI2 with tissue annotations as pre sented in section 7.1.1.
- **Lesion data S_lesion_:** We evaluate our method on the training set of BraTS18 (Menze et al., 2015; Bakas et al., 0000) which con tains the scans of 285 patients, 210 with high grade glioma and 75 with low grade glioma. 129 patients have a tumour located in one hemisphere only. Four scans (*T*_1_, *T*_1_ c, *T*_2_ and FLAIR) have been acquired for each patient and pre-processed by the or ganisers: Co-registration, skull-stripping and re-sampling to an isotropic 1mm resolution. Manual annotations include three tu mour labels: 1) Necrotic core and non-enhancing tumour; 2) oedema; and 3) enhancing core.

The acquisition protocols of the *T*_1_ scans in the two datasets are inconsistent. Specifically, MP-RAGE was used for the tissue data **S_control_**, while we observed other protocols such as fast spin echo (SE) for **S_lesion_**. Note that the detailled acquisition settings for **S_lesion_** are not publicly available.

#### 7.2.2 Description of the compared models

In order to evaluate our framework with and without the domain adaptation (DA) component, different models are considered, as presented in 5.

- **Pipeline model (*Pipeline*)**: This model corresponds to the com bination of two task-specific models:
  A *Tissue* segmentation model that only performs tissue segmentation and is trained on the *T*_1_ scans from the dataset with tissue annotations **S_control_**.
  A *Lesion* model that only performs lesion segmentation and is trained using the *T*_1_, *T*_1_ c, *T*_2_ and FLAIR scans from the dataset with lesion annotations **S_lesion_**.
- **Proposed joint model without DA (*jSTABL*)**: Our joint Segmen tation Tissue And Brain Lesion model is trained using our train ing procedure without domain adaptation, tissue segmentation is learned from the *T*_1_ scans in **S_control_**.
- **jSTABL + data augmentation (*jSTABL+Augm*)**: Corresponds to our *jSTABL* model with DA based on data augmentation.
- **jSTABL + adversarial DA (*jSTABL+Adv*)**: Corresponds to our *jSTABL* model with DA based on adversarial learning.
- **jSTABL + 5 synthetic control scans (*jSTABL+5*)**: Corresponds to our *jSTABL* model with only 5 additional pseudo-healthy scans.
- **jSTABL + 90 synthetic control scans (*jSTABL+90*)**: Corresponds to our *jSTABL* model with 90 additional pseudo-healthy scans.

Note that the pseudo-healthy scans used for training were generated from the training *lesion* scans to avoid introducing bias at testing stage.

In this set of experiments, the skull-stripped images are first cropped to remove the blank spaces and then random patches of size (112,112,112) are fed to the network.

#### 7.2.3 Method for assessing the models

While the evaluation of the tumour segmentation on BraTS18 and the tissue segmentation on the *control* data (OASIS1+ADNI2) is straightforward using the manual annotations, the tissue segmentation performance cannot be assessed on BraTS18 due to the missing tissue annotations. For this reason we propose two methods to assess quantitatively and qualitatively the tissue segmentation on BraTS18.

*Quantitative assessment using the symmetrised data* Firstly, we propose to use the 129 patients from BraTS18 with a tumour located in one side to generate 129 pseudo-healthy symmetrised data with the bronze standard tissue annotations from GIF as ground truth. Examples are shown in Fig. C.9. By computing the Dice Score Coefficient between the predictions on the symmetrised BraTS18 data and the bronze standard ground truth, we quantitatively evaluate our model on the pseudo-healthy hemisphere of BraTS18 samples.

*Qualitative assessment on anatomical landmarks* Secondly, in order to assess the accuracy of the models on the tissues surrounding the tumour, we propose a new qualitative protocol. This protocol is based on the Alberta Stroke Program Early CT Score (ASPECTS) which was originally proposed to assess early ischaemic cerebral changes on CT or MRI scans (Barber et al., 2000). The stroke scores are obtained by assessing the integrity of 10 anatomical landmarks as shown on 8. Scores and associated template are commonly used in clinical practice.

The landmarks were chosen because they are easily identifiable, reliable amongst readers and capture a large cerebral coverage. The landmarks include or delineate our tissue classes of interest: Grey matter; white matter; basal ganglia; and ventricles. Instead of evaluating loss of clarity of landmarks due to ischemia we evaluated loss of clarity of landmarks due to incorrect tissue predictions. Unlike ASPECTS, which excludes infratentorial structures which are difficult to evaluate on CT, we added the brainstem and the cerebellum as two additional landmarks for a total of 12 anatomical landmarks. We named our assessment method Anatomy ASPECTS+. For each landmark, 3 scores are possible: 0 = anatomy inaccurate; 0.5 = anatomy mostly accurate; and 1 = anatomy highly accurate. Anatomical landmarks that were infiltrated with substantial tumour were excluded.

For our experiments, we randomly drew 20 patients from the testing sets of BraTS18 and two senior neuro-radiologists independently evaluated the quality of the predictions using Anatomy ASPECTS+ for 4 methods: 1) *Les. Ann. + GIF* pipeline that uses the tumour annotations and tissue segmentation obtained by GIF while the tumour is masked; 2) *Pipeline*; 2) *jSTABL*; 3) *jSTABL+5*; 4) *jSTABL+10*. The neuro-radiologists assessed blindly the 4 methods, in a randomised order, for the 20 patients.

#### 7.2.4 Results

Table 4 shows the DSC for the 6 tissue classes and the three tumour classes on BraTs18.

**Fig. 6:**
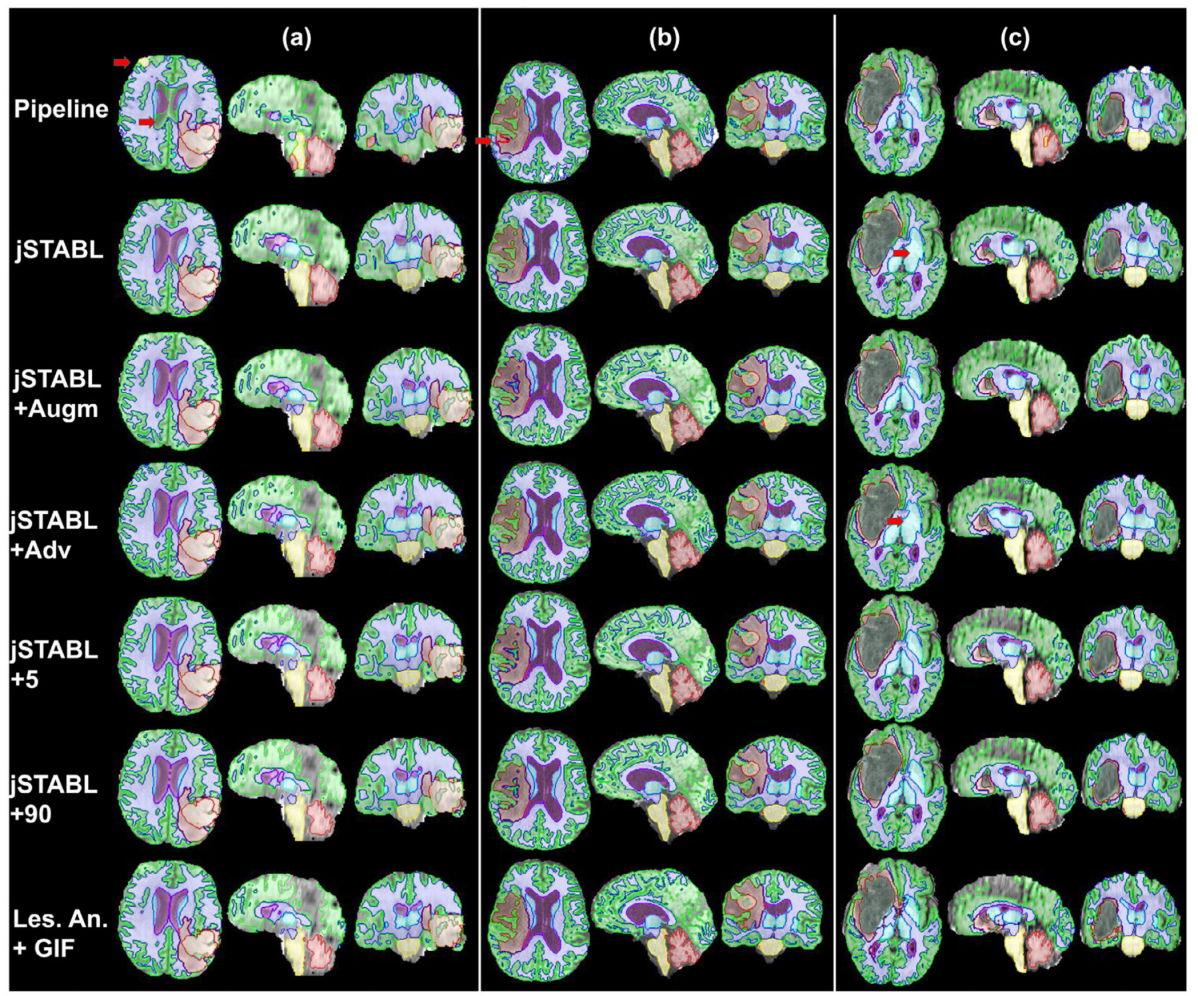
Examples of multi-modal outputs shown on T_1_ scans from BraTS18 for the different models: *Pipeline, jSTABL, jSTABL+Augm, jSTABL+Adv, jSTABL+5, jSTABL+90* and *Les. Ann. + GIF* pipeline. Scans with different resolutions, contrasts and grades (High Grade for (a) and (b), Low Grade for (c)) are presented.

Firstly, *jSTABL* model outperforms *Pipeline* on tissue segmentation. We observed that the presence of a large tumour creates major perturbations for the *Tissue* model. For example, we found samples for which the tumour and the surrounding tissues were partially classified as cerebellum, even though the tumour was far from the cerebellum, as shown in Fig. 6. In contrast, such artefacts were not observed for *jSTABL* model, demonstrating again advantages of our method compared to a simpler *Pipeline* approach.

Secondly, while obtaining relatively good performance on most of the tissue classes, *jSTABL* model fails to segment correctly grey matter and basal ganglial. This highlights the needs for domain adaptation.

Thirdly, learning from pseudo-healthy annotated scans (*jSTABL+5* and *jSTABL+90*) outperforms the other unsupervised DA strategies based either on data augmentation (*jSTABL+Augm*) and adversarial learning (*jSTABL+Adv*). This demonstrates the benefits of using a supervised approach for our problem. Moreover, only 5 pseudo-healthy annotated scans are required to obtain an accuracy similar to the one on the *control* data (see Table 2), i.e. to bridge the domain gap. Fig. 6 shows that learning from pseudo-healthy annotated scans allows the network to be robust to variations in resolution, contrast or glioma grade, even with few samples used for domain adaptation.

**Fig. 7:**
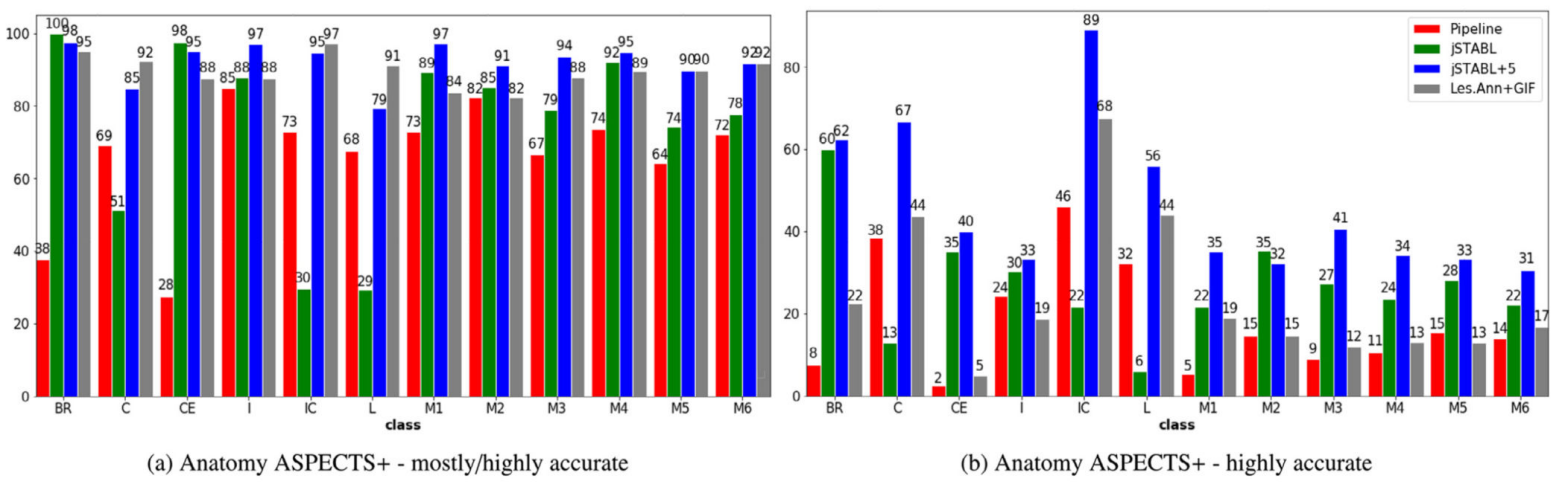
Comparison of our method (jSTABL) with the GIF framework using the proposed Anatomy ASPECTS+ qualitative assessment methodology. BR = Brainstem, C = Caudate, CE = Cerebellum, I = Insula, IC = Internal Capsule, L = Lentiform Nucleus, M1 = Frontal operculum, M2 = Anterior temporal lobe, M3 = Posterior temporal lobe, M4 = Anterior MCA, M5 = Lateral MCA, M6 = Posterior MCA.

Finally, Anatomy ASPECTS+ is employed to provide a quantitative assessment of the segmentation of the tissues surrounding the tumour. Four models are compared by two neuro-radiologists: *Pipeline, jSTABL, jSTABL+5* and the time-consuming *Les. Ann. + GIF* pipeline that requires manual annotations of the lesions. Results are presented in Fig. 7. First, *jSTABL* is more often “mostly accurate” than the *Pipeline* (mean score - 75% vs 66%). Again, this highlights the strength of our joint model compared to a pipeline approach. Secondly, *jSTABL+5* is more often “highly accurate” than the *Les. Ann. + GIF* pipeline (mean score - 46% vs 24%). This shows that our fast and fully automatic method can be considered as a new state-of-the-art for performing joint tissue and lesion segmentation.

## 8 Discussion

In this section, we discuss some of the limitations of the different methods.

Firstly, a common modality across the task-specific dataset is required to transfer the knowledge learn between the task-specific sets of modality. Without this common modality, the upper bound is not tractable anymore and our method cannot be applied.

Secondly, our approach relies on a simple hetero-modal architecture that aims to encode modalities in a common shared feature space. Ye t, averaging the feature maps doesn’t enforce the network to learn a shared feature representation. To tackle this problem, a hetero-modal variational auto-encoder architecture has been recently introduced Dorent et al. (2019a). Based on a principled formulation of the problem, the induced loss function is the cross-entropy, which does not satisfy the triangle inequality. Consequently, without further research, this approach cannot be directly integrated in the formulation of our problem.

Thirdly, we found that the presence of lesions does not always perturbed a network trained on *control* data, especially for small lesions. Consequently, our method didn’t always show large improvements compared to a simpler *Pipeline* approach on WMH. However, we observed that *Pipeline* can be perturbed by larger pathology and thus is less robust.

## 9 Conclusion

This work addresses the challenge of learning a joint brain tissue and lesion segmentation with disjoint heterogeneous annotations. Our novel approach is mathematically grounded, conceptually simple, and relies on reasonable assumptions.

The main contribution of this work is to overcome the challenge of the lack of fully-annotated data for joint problems. We demonstrate that a model trained on databases providing either the tissue or the lesion annotations and with different modalities can achieve similar performance to a model trained on a fully-annotated joint dataset. Our work also shows that the knowledge learnt from one modality can be preserved when more modalities are used as input. Finally, domain adaptation for image segmentation can be performed with a small set of data related to the target distribution.

In the future, we will evaluate our approach on new datasets with other lesions. Furthermore, we would like to extend our method to include the full parcellation of the brain (143 structures). Finally, we plan to integrate uncertainty measures in our framework as a future work. As one of the first work to methodologically address the problem of joint learning from hetero-modal and domain-shifted datasets, we believe that our approach will help DNN make further impact in clinical scenarios.

## Supplementary Material

Appendix

## Figures and Tables

**Fig. 8:**
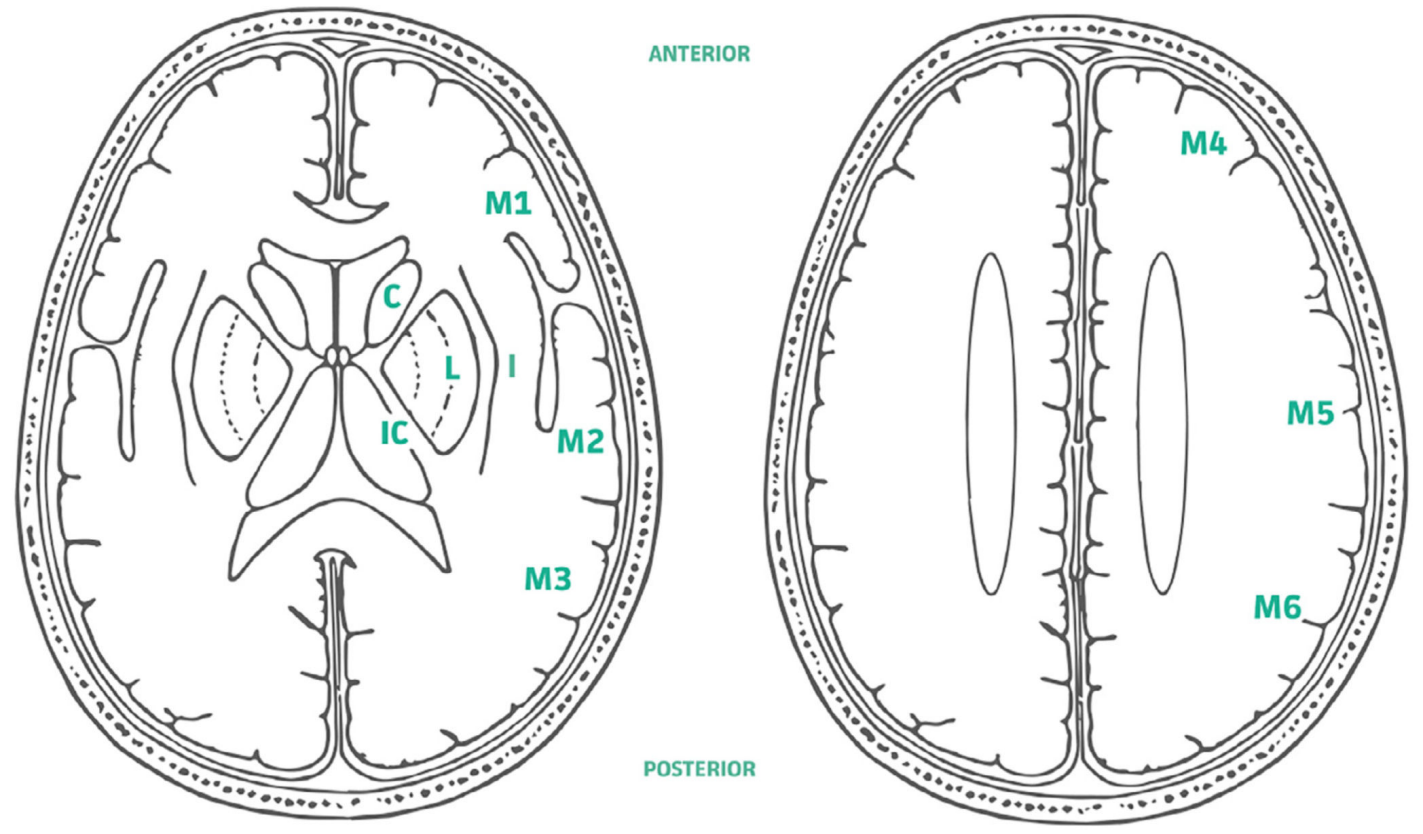
ASPECTS anatomical landmarks (Barber et al., 20 0 0) used in our qualitative assessment methodology in the absence of joint ground truth. C = Caudate, I = Insula, IC = Internal Capsule, L = Lentiform Nucleus, M1 = Frontal operculum, M2 = Anterior temporal lobe, M3 = Posterior temporal lobe, M4 = Anterior MCA, M5 = Lateral MCA, M6 = Posterior MCA Illustration courtesy of P.A. Barber.

**Table 1:** Summary of data characteristics for white matter lesion segmentation.

	Control data		Lesion data			Fully anno. data
	OASIS1 (Marcus et al., 2007)	ADNI2 (Jack Jr. et al., 2008)	WMH-Utrech (Kuijf et al., 2019)	WMH-Singapore	WMH-Amsterdam	MRBrainS18 (Kuijf and Bennink, 2018)
Sequences	3D MP-RAGE T_1_	3D MP-RAGE T_1_	3D MP-RAGE T_1_	3D MP-RAGE T_1_	3D MP-RAGE T_1_	MP-RAGE 3D T_1_
	×	×	2D FLAIR	2D FLAIR	3D FLAIR	2D FLAIR
MRI scanner	Siemens Vision	Various	Philips Achieva	Siemens TrioTim	GE Signa HDxt	Philips Achieva
Field Strengh	1.5T	3T	3T	3T	3T	3T
Voxel size (mm^3^)	1.00 × 1.00 × 1.00	1.20 × 1.05 × 1.05	0.96 × 0.95 × 3.00 WMH	1.00 × 1.00 × 3.00 WMH	1.20 × 0.98 × 3.00 WMH	0.96 × 0.96 × 3.00
Annotations	143 structures	143 structures	WMH	WMH	WMH	6 Tissues + WMH+CSF
# scans available	35	25	20	20	20	30 (7 available)
Training data in:	*Tissue + jSTABL*	*Tissue + jSTABL*	*Lesion + jSTABL*	*Lesion + jSTABL*	*Lesion + jSTABL*	*Fully*

**Table 2:** Evaluation of our framework (jSTABL) on patients with White Matter Lesion in comparison with baseline methods. We report means and standard deviations for Dice scores. Means only are reported in the online leader-board, leading to missing standard deviations.

Classes	OASIS1 + ADNI2			WMH			MRBrainS18			
	Tissue	Fully-Sup	jSTABL	Pipeline	Fully-Sup	jSTABL	SPM	Pipeline	Fully-Sup	jSTABL
Grey matter	88.3 (3.4)	81.6 (2.5)	88.3 (3.4)	88.3 (2.1)	85.4 (2.7)	**88.8 (2.1)**	76.5	82.3	83.7	82.2
White mater	92.8 (2.3)	83.3 (2.6)	92.3 (2.7)	92.1 (1.8)	85.4 (2.6)	**92.4 (1.5)**	75.7	85.0	85.7	85.6
Brainstem	93.5 (1.0)	71.7 (2.7)	93.0 (0.9)	93.6 (1.0)	77.1 (2.4)	**94.2 (0.9)**	76.5	72.8	85.0	73.3
Basal ganglia	89.5 (3.0)	69.6 (4.1)	88.4 (2.7)	**86.3 (4.3)**	74.2 (2.0)	85.1 (3.4)	74.7	77.4	79.7	78.0
Ventricles	90.3 (4.3)	70.5 (18.0)	90.6 (3.8)	94.7 (2.3)	92.1 (4.2)	**95.7 (1.4)**	80.9	91.8	92.2	92.9
Cerebellum	95.0 (1.2)	92.0 (1.4)	94.9 (1.1)	95.7 (1.0)	93.8 (2.0)	**96.0 (0.9)**	89.4	89.2	93.2	90.4
White matter Lesion				77.4 (9.6)	60.1 (19.1)	**77.6 (9.2)**	40.8	58.4	56.2	59.4

**Table 3:** Evaluation of our framework (jSTABL) on patients with White Matter Lesion in comparison with baseline methods. We report means and standard deviations for 95th-percentile Hausdorff distances. Means only are reported in the online leader-board, leading to missing standard deviations.

Classes	OASIS1 + ADNI2			WMH			MRBrainS18			
	Tissue	Fully-Sup	jSTABL	Pipeline	Fully-Sup	jSTABL	SPM	Pipeline	Fully-Sup	jSTABL
Grey matter	1.3 (0.4)	2.0 (0.4)	1.4 (0.5)	1.2 (0.2)	1.3 (0.3)	**1.1 (0.2)**	2.9	1.9	1.9	2.1
White mater	1.2 (0.5)	3.0 (0.1)	1.2 (0.5)	**1.1 (0.2)**	2.0 (0.3)	**1.1 (0.1)**	4.9	3.2	2.9	3.2
Brainstem	1.4 (0.4)	10.5 (2.3)	1.7 (0.4)	1.7 (0.4)	8.8 (2.2)	**1.3 (0.4)**	25.3	11.6	6.65	11.6
Basal ganglia	1.8 (0.5)	4.9 (0.8)	2.1 (0.3)	**2.0 (0.6)**	3.6 (0.4)	2.5 (0.3)	7.1	4.3	4.3	4.0
Ventricles	1.9 (2.5)	20.4 (12.2)	1.8 (2.5)	1.4 (1.2)	5.1 (10.5)	**1.0 (0.1)**	5.8	3.2	3.0	2.9
Cerebellum	2.3 (0.6)	3.3 (0.4)	2.4 (0.6)	1.8 (0.7)	3.2 (1.7)	**1.8 (0.6)**	4.3	5.1	3.7	4.8
White matter Lesion				4.6 (3.8)	11.0 (9.4)	**4.2 (3.6)**	25.3	10.2	13.3	7.2

**Table 4:** Evaluation of our framework (jSTABL) on patients with gliomas in comparison to baseline methods. We report means and standard deviations for Dice scores. Metrics were computed on the BraTS 2018 validation dataset.

Models	w/o DA		w/ DA		w/ pseudo-healthy gen.	
	Pipeline	jSTABL	jSTABL + Adv	jSTABL + Augm	jSTABL + 5	jSTABL + 90
Grey Matter	76.1 (8.4)	79.1 (5.2)	81.1 (4.6)	82.8 (4.7)	88.3 (3.9)	88.8 (4.0)
White Matter	85.4 (5.7)	87.0 (4.4)	88.1 (4.4)	90.3 (2.8)	93.1 (2.5)	93.3 (2.6)
Brainstem	81.5 (17.6)	92.4 (2.5)	92.6 (1.9)	92.4 (2.0)	94.9 (1.4)	95.5 (1.5)
Basal Ganglia	72.7 (20.1)	73.1 (7.3)	77.7 (7.1)	84.7 (5.1)	89.7 (3.5)	90.5 (3.2)
Ventricles	75.0 (26.9)	91.8 (6.4)	92.5 (4.5)	93.4 (4.4)	94.7 (4.2)	95.1 (3.8)
Cerebellum	86.5 (11.3)	93.2 (4.6)	93.7 (3.5)	94.0 (2.4)	94.7 (4.5)	95.1 (2.9)
Whole Tumour	87.9 (8.7)	88.1 (6.7)	87.7 (8.1)	88.3 (9.0)	88.2 (8.1)	88.1 (9.4)
Core Tumour	78.6 (20.5)	79.1 (19.6)	79.5 (18.9)	80.4 (18.6)	80.5 (18.1)	80.9 (17.9)
Enhancing Tumour	69.9 (29.1)	70.0 (28.6)	70.0 (28.9)	71.4 (27.7)	70.3 (28.8)	71.0 (28.3)

## References

[R1] Ashburner J, Friston KJ (2000). Voxel-based morphometry – the methods. Neuroimage.

[R2] Bakas S, Reyes M Identifying the Best Machine Learning Algorithms for Brain Tumor Segmentation, Progression Assessment, and Overall Survival Prediction in the BRATS Challenge.

[R3] Barber PA, Demchuk AM, Zhang J, Buchan AM (2000). Validity and reliability of a quantitative computed tomography score in predicting outcome of hyperacute stroke before thrombolytic therapy. The Lancet.

[R4] Battaglini M, Jenkinson M, De Stefano N (2012). Evaluating and reducing the impact of white matter lesions on brain volume measurements. Hum Brain Mapp.

[R5] Ben-David S, Blitzer J, Crammer K, Kulesza A, Pereira F, Vaughan JW (2010). A theory of learning from different domains. Mach Learn.

[R6] Bermel RA, Bakshi R (2006). The measurement and clinical relevance of brain atrophy in multiple sclerosis. The Lancet Neurology.

[R7] Bilen H, Vedaldi A (2016). Weakly supervised deep detection networks. The IEEE Conference on Computer Vision and Pattern Recognition (CVPR).

[R8] Bitar R, Leung G, Perng R, Tadros S, Moody AR, Sarrazin J, McGregor C, Christakis M, Symons S, Nelson A, Roberts TP (2006). MR Pulse sequences: what every radiologist wants to know but is afraid to ask. Radiographics.

[R9] Bottou L, Curtis FE, Nocedal J (2018). Optimization methods for large-scale machine learning. SIAM Rev.

[R10] Bragman FJS, Tanno R, Eaton-Rosen Z, Li W, Hawkes DJ, Ourselin S, Alexander DC, McClelland JR, Cardoso MJ (2018). Uncertainty in multitask learning: Joint representations for probabilistic MR-only radiotherapy planning.

[R11] Cardoso MJ, Modat M, Wolz R, Melbourne A, Cash D, Rueckert D, Ourselin S (2015). Geodesic information flows: spatially-variant graphs and their application to segmentation and fusion. IEEE Trans Med Imaging.

[R12] Chen S, Bortsova G, García-Uceda Juárez A, van Tulder G, de Bruijne M, Shen D, Liu T, Peters TM, Staib LH, Essert C, Zhou S, Ya p P-T, Khan A (2019). Multi-task attention-based semi-supervised learning for medical image segmentation. Medical Image Computing and Computer Assisted Intervention – MICCAI 2019.

[R13] Chen X, Konukoglu E (2018). Unsupervised detection of lesions in brain MRI using constrained adversarial auto-encoders.

[R14] Çiçek Ö, Abdulkadir A, Lienkamp SS, Brox T, Ronneberger O, Ourselin S, Joskowicz L, Sabuncu MR, Unal G, Wells W (2016). 3d u-net: Learning dense volumetric segmentation from sparse annotation. Medical Image Computing and Computer-Assisted Intervention – MICCAI 2016.

[R15] Çiçek Ö, Abdulkadir A, Lienkamp SS, Brox T, Ronneberger O, Ourselin S, Joskowicz L, Sabuncu MR, Unal G, Wells W (2016). 3d u-net: Learning dense volumetric segmentation from sparse annotation. Medical Image Computing and Computer-Assisted Intervention – MICCAI 2016.

[R16] Csurka G (2017). A Comprehensive Survey on Domain Adaptation for Visual Applications. Domain Adaptation in Computer Vision Applications.

[R17] Dorent R, Joutard S, Modat M, Ourselin S, Vercauteren T (2019). Hetero-modal variational encoder-decoder for joint modality completion and segmentation.

[R18] Dorent R, Li W, Ekanayake J, Ourselin S, Vercauteren T (2019). Learning joint lesion and tissue segmentation from task-specific hetero-modal datasets.

[R19] Dou Q, Ouyang C, Chen C, Chen H, Heng P-A (2018). Unsupervised cross-modality domain adaptation of convnets for biomedical image segmentations with adversarial loss. In: Proceedings of the Twenty-Seventh International Joint Conference on Artificial Intelligence, IJCAI-18.

[R20] Dwyer MG, Bergsland N, Ramasamy DP, Jakimovski D, Weinstock-Guttman B, Zivadinov R (2018). Atrophied brain lesion volume: a new imaging biomarker in multiple sclerosis. Journal of Neuroimaging.

[R21] Fisniku LK, Chard DT, Jackson JS, Anderson VM, Altmann DR, Miszkiel KA, Thompson AJ, Miller DH (2008). Gray matter atrophy is related to long-term disability in multiple sclerosis. Ann Neurol.

[R22] Geurts JJ, Calabrese M, Fisher E, Rudick RA (2012). Measurement and clinical effect of grey matter pathology in multiple sclerosis. The Lancet Neurology.

[R23] Giorgio A, De Stefano N (2013). Clinical use of brain volumetry. J Magn Reson Imaging.

[R24] Havaei M, Guizard N, Chapados N, Bengio Y (2016). HeMIS: Hetero-modal image segmentation.

[R25] He K, Zhang X, Ren S, Sun J (2015). Delving deep into rectifiers: Surpassing human-level performance on ImageNet classification.

[R26] Iglesias JE, Liu C, Thompson PM, Tu Z (2011). Robust brain extraction across datasets and comparison with publicly available methods. IEEE Trans Med Imaging.

[R27] Iglesias JE, Sabuncu MR (2015). Multi-atlas segmentation of biomedical images: a survey. Med Image Anal.

[R28] Isensee F, Petersen J, Kohl SAA, Jäger PF, Maier-Hein KH (2019). Nnu-net: breaking the spell on successful medical image segmentation.

[R29] Jack CR, Bernstein MA, Fox NC, Thompson P, Alexander G, Harvey D, Borowski B, Britson PJL, Whitwell J, Ward C, Dale AM (2008). The alzheimer’s disease neuroimaging initiative (adni): mri methods. J Magn Reson Imaging.

[R30] Jenkinson M, Beckmann CF, Behrens TE, Woolrich MW, Smith SM (2012). FSL. Neuroimage.

[R31] Kamnitsas K, Baumgartner C, Ledig C, Newcombe V, Simpson J, Kane A, Menon D, Nori A, Criminisi A, Rueckert D (2017). Unsupervised domain adaptation in brain lesion segmentation with adversarial networks.

[R32] Kendall A, Gal Y (2017). What uncertainties do we need in Bayesian deep learning for computer vision?. Proceedings of Advances in Neural Information Processing Systems 30 (NIPS 2017).

[R33] Kim D-J, Choi J, Oh T-H, Yoon Y, Kweon IS (2018). Disjoint multi-task learning between heterogeneous human-centric tasks.

[R34] Kingma DP, Ba J (2015). Adam: A method for stochastic optimization.

[R35] Kosub S (2018). A note on the triangle inequality for the jaccard distance. Pattern Recognit Lett.

[R36] Kuijf HJ, Bennink E (2018). MRBrainS18. http://mrbrains18.isi.uu.nl/.

[R37] Kuijf HJ, Biesbroek JM, de Bresser J, Heinen R, Andermatt S, Bento M, Berseth M, Belyaev M, Cardoso MJ, Casamitjana A, Collins DL (2019). Standardized assessment of automatic segmentation of white matter hyperintensities; results of the WMH segmentation challenge. IEEE Trans Med Imaging.

[R38] Le T-L-T, Thome N, Bernard S, Bismuth V, Patoureaux F, Cardoso MJ, Feragen A, Glocker B, Konukoglu E, Oguz I, Unal G, Vercauteren T (2019). Multitask classification and segmentation for cancer diagnosis in mammography.

[R39] Li W, Wang G, Fidon L, Ourselin S, Cardoso MJ, Vercauteren T (2017). On the compactness, efficiency, and representation of 3D convolutional networks: Brain parcellation as a pretext task. Proceedings of Information Processing in Medical Imaging (IPMI’17).

[R40] Li Z, Hoiem D (2017). Learning without forgetting. IEEE Trans Pattern Anal Mach Intell.

[R41] Long M, Cao Y, Wang J, Jordan M, Bach F, Blei D (2015). Learning transferable features with deep adaptation networks. Proceedings of the 32nd International Conference on Machine Learning.

[R42] Long M, CAO Z, Wang J, Jordan MI, Bengio S, Wallach H, Larochelle H, Grauman K, Cesa-Bianchi N, Garnett R (2018). Conditional Adversarial Domain Adaptation. Advances in Neural Information Processing Systems 31.

[R43] Maillard P, Carmichael O, Harvey D, Fletcher E, Reed B, Mungas D, DeCarli C (2013). FLAIR And diffusion MRI signals are independent predictors of white matter hyperintensities. American Journal of Neuroradiology.

[R44] Marcus DS, Wang TH, Parker J, Csernansky JG, Morris JC, Buckner RL (2007). Open access series of imaging studies (OASIS): cross-sectional MRI data in young, middle aged, nondemented, and demented older adults. J Cogn Neu-rosci.

[R45] Menze BH, Jakab A, Bauer S, Kalpathy-Cramer J, Farahani K, Kirby J, Burren Y, Porz N, Slotboom J, Wiest R, Lanczi L (2015). The multimodal brain tumor image segmentation benchmark (BRATS). IEEE Trans Med Imaging.

[R46] Moeskops P, de Bresser J, Kuijf HJ, Mendrik AM, Biessels GJ, Pluim JP, Isgum I (2018). Evaluation of a deep learning approach for the segmentation of brain tissues and white matter hyperintensities of presumed vascular origin in MRI. NeuroImage: Clinical.

[R47] Moeskops P, Wolterink JM, van der Velden BHM, Gilhuijs KGA, Leiner T, Viergever MA, Išgum I, Ourselin S, Joskowicz L, Sabuncu MR, Unal G, Wells W (2016). Deep learning for multi-task medical image segmentation in multiple modalities. Medical Image Computing and Computer-Assisted Intervention – MICCAI 2016.

[R48] Oquab M, Bottou L, Laptev I, Sivic J (2015). Is object localization for free? - Weakly-supervised learning with convolutional neural networks. The IEEE Conference on Computer Vision and Pattern Recognition (CVPR).

[R49] Orbes-Arteaga M, Varsavsky T, Sudre CH, Eaton-Rosen Z, Haddow LJ, Sørensen L, Nielsen M, Pai A, Ourselin S, Modat M, Nachev P (2019). Multi-domain adaptation in brain mri through paired consistency and adversarial learning. DART/MIL3ID@MICCAI.

[R50] Pan SJ, Tsang IW, Kwok JT, Yang Q (2011). Domain adaptation via transfer component analysis. IEEE Trans Neural Networks.

[R51] Pérez-García F, Sparks R, Ourselin S (2020). Torchio: a python library for efficient loading, preprocessing, augmentation and patch-based sampling of medical images in deep learning.

[R52] Popescu V, Agosta F, Hulst HE, Sluimer IC, Knol DL, Sormani MP, Enzinger C, Ropele S, Alonso J, Sastre-Garriga J, Rovira A (2013). Brain atrophy and lesion load predict long term disability in multiple sclerosis. Journal of Neurology, Neuro-surgery & Psychiatry.

[R53] Prados F, Cardoso MJ, Kanber B, Ciccarelli O, Kapoor R, Wheeler-Kingshott CAG, Ourselin S (2016). A multi-time-point modality-agnostic patch-based method for lesion filling in multiple sclerosis. Neuroimage.

[R54] Prima S, Ourselin S, Ayache N (2002). Computation of the mid-sagittal plane in 3-D brain images. IEEE Trans Med Imaging.

[R55] Roulet N, Slezak DF, Ferrante E (2019). Joint learning of brain lesion and anatomy segmentation from heterogeneous datasets.

[R56] Ruder S (2017). An overview of multi-task learning in deep neural networks.

[R57] Saha O, Sathish R, Sheet D, Cardoso MJ, Feragen A, Glocker B, Konukoglu E, Oguz I, Unal G, Vercauteren T (2019). Learning with multitask adversaries using weakly labelled data for semantic segmentation in retinal images. Proceedings of The 2nd International Conference on Medical Imaging with Deep Learning.

[R58] Sdika M, Pelletier D (2009). Nonrigid registration of multiple sclerosis brain images using lesion inpainting for morphometry or lesion mapping. Hum Brain Mapp.

[R59] Shaw R, Sudre C, Ourselin S, Cardoso MJ, Cardoso MJ, Feragen A, Glocker B, Konukoglu E, Oguz I, Unal G, Vercauteren T (2019). Mri k-space motion artefact augmentation: Model robustness and task-specific uncertainty. Proceedings of The 2nd International Conference on Medical Imaging with Deep Learning.

[R60] Simpson AL, Antonelli M, Bakas S, Bilello M, Farahani K, van Ginneken B, Kopp-Schneider A, Landman BA, Litjens GJS, Menze BH, Ronneberger O (2019). A large annotated medical image dataset for the development and evaluation of segmentation algorithms.

[R61] Späth H (1981). The minisum location problem for the jaccard metric. Operations-Research-Spektrum.

[R62] Sudre CH, Cardoso MJ, Ourselin S (2017). Longitudinal segmentation of age-related white matter hyperintensities. Med Image Anal.

[R63] Sun B, Feng J, Saenko K (2016). Return of frustratingly easy domain adaptation. Proceedings of the Thirtieth AAAI Conference on Artificial Intelligence.

[R64] Sun L, Wang J, Ding X, Huang Y, Paisley JW (2018). An adversarial learning approach to medical image synthesis for lesion removal.

[R65] Ulyanov D, Vedaldi A, Lempitsky VS (2016). Instance normalization: The missing ingredient for fast stylization.

[R66] Valverde S, Oliver A, Lladó X (2014). A white matter lesion-filling approach to improve brain tissue volume measurements. NeuroImage: Clinical.

[R67] Wen PY, Macdonald DR, Reardon DA, Cloughesy TF, Sorensen AG, Galanis E, DeGroot J, Wick W, Gilbert MR, Lassman AB, Tsien C (2010). Updated response assessment criteria for high-grade gliomas: response assessment in neuro-oncology working group. Journal of Clinical Oncology.

[R68] Xia T, Chartsias A, Tsaftaris SA, Cardoso MJ, Feragen A, Glocker B, Konukoglu E, Oguz I, Unal G, Vercauteren T (2019). Adversarial pseudo healthy synthesis needs pathology factorization. Proceedings of The 2nd International Conference on Medical Imaging with Deep Learning.

[R69] Xu Y, Zhu J-Y, Chang EI-C, Lai M, Tu Z (2014). Weakly supervised histopathol-ogy cancer image segmentation and classification. Med Image Anal.

[R70] Zhang Y, Yang Q (2017). A survey on multi-task learning.

[R71] Zhao H, des Combes RT, Zhang K, Gordon GJ (2019). On learning invariant representations for domain adaptation. Proceedings of the 36th International Conference on Machine Learning, ICML 2019, 9–15 June 2019.

