# Supplementary material for: Learning joint segmentation of tissues and brain lesions from task-specific hetero-modal domain-shifted datasets
Source: Med Image Anal. Author manuscript; Available in PMC 2021 Mar 4. (PMC7116853; doi:10.1016/j.media.2020.101862)
Supplement: Appendix [file EMS117272-supplement-Appendix.pdf]

Appendix A. Probabilistic multi-class Jaccard loss function

First we recall the definitions of the binary Jaccard distance and the proposed extension to probabilistic inputs. The binary Jaccard distance J_{bin} is defined such that:

$$\forall a, b \in \{0,1\}^N,\ J_{bin}(a,b) = 1 - \frac{\sum_{i=1}^{N} a_i b_i}{\sum_{i=1}^{N} a_i + b_i - a_i b_i} \quad \text{(A.1)}$$

Definition A.1. (Probabilistic multi-class Jaccard distance)

Let C be the number of classes in $\mathcal{C}$, N be the number of voxels and $\mathcal{P} \subset [0,1]^{C\times N}$ denote the set of probability vector map such that for any $p = (p_{c,i})_{c\in\mathcal{C}, i\in[0;N]} \in \mathcal{P}$:

$$\forall i \in [0;N],\ \sum_{c\in C} p_{c,i} = 1$$

The probabilistic multi-class Jaccard distance is defined for any $(u,v) \in \mathcal{P}^2$ as:

$$\mathcal{J}(u,v) = \sum_{c\in C} \omega_c \underbrace{\frac{2\sum_{i=1}^{N} |u_{c,i} - v_{c,i}|}{\sum_{i=1}^{N} |u_{c,i}| + |v_{c,i}| + |u_{c,i} - v_{c,i}|}}_{\mathcal{J}_c} \quad \text{(A.2)}$$

where ω_c are class-specific weights summing up to one.

A1. Relation between the probabilistic jaccard loss and the binary jaccard distance

The binary case corresponds to a two-class problem, i.e. $\mathcal{C} = \{0,1\}$. Let $a,\ b \in \{0,\ 1\}^N$ be two binary vectors of size n and let u and v denote respectively the categorical encodings of

a and b, i.e. $a_i = 1 \iff (u_{1,i} = 1 \text{ and } u_{0,i} = 0)$ and $b_i = 1 \iff (v_{1,i} = 1 \text{ and } v_{0,i} = 0)$. The binary Jaccard distance can be rewritten as:

$$\mathcal{J}_{bin}(a,b) = 1 - \frac{\sum_{i=1}^N a_i b_i}{\sum_{i=1}^N a_i + b_i - a_i b_i} = \frac{\sum_{i=1}^N a_i + b_i - 2a_i b_i}{\sum_{i=1}^N a_i + b_i - a_i b_i}$$

Given that for all $i \in \{1,\ldots,N\}$, $a_i = 0$ or $a_i = 1$, we observe that $a_i^2 = a_i$. Using the same property for b, we get:

$$J_{bin}(a,b) = \frac{\sum_{i=1}^N (a_i - b_i)^2}{\sum_{i=1}^N a_i^2 + b_i^2 - a_i b_i} = \frac{2\sum_{i=1}^N (a_i - b_i)^2}{\sum_{i=1}^N a_i^2 + b_i^2 + (a_i - b_i)^2}$$

Finally, given that for all $i \in \{1,\ldots,N\}$, $(a_i - b_i)^2 = 0$ or $(a_i - b_i)^2 = 1$, we have that $(a_i - b_i)^2 = |a_i - b_i|$ and conclude that:

$$\mathcal{J}_{bin}(a,b) = \frac{2\sum_{i=1}^N |a_i - b_i|}{\sum_{i=1}^N a_i^2 + b_i^2 + |a_i - b_i|} = \mathcal{J}_1(u,v)$$

A2. Proof of lemma 4.1

The proof that the probabilistic Jaccard is a distance is based on the Steinhaus transform (Späth, 1981). Given a metric space (E, d) with a distance d and given a fixed point $\alpha \in E$, we can define a new distance d_{new} as:

$$d_{new}(x,y) = \frac{d(x,y)}{d(x,\alpha) + d(y,\alpha) + d(x,y)}$$

Consequently, the probabilistic Jaccard loss distance $\mathcal{J}_c$ defined in (A.2):

$$\forall u, v \in [0,1]^{C\times N},\ \ \mathcal{J}_c(u,v) = \frac{2\|u_c - v_c\|_1}{\|u_c\|_1 + \|v_c\|_1 + \|u_c - v_c\|_1} \tag{A.3}$$

can be seen as a Steinhaus transform of the metric space $([0,1]^N, ||.||_1)$ with $\alpha = 0$ and thus is a distance. Given that the weighted sum of distances is a distance, we finally conclude that the probabilistic multi-class Jaccard defined as:

$$\mathcal{J} = \sum_{c\in C} \mathcal{J}_c \tag{A.4}$$

is a distance.

Appendix B. Proof of Proposition 1

First, Eqs. (3), (9) and (7) are combined:

$$\begin{aligned}\mathbb{E}_{\mathcal{D}_{lesion}}[\mathcal{L}(h_\theta(x), y)] &\le \mathcal{R}_{seg} + \epsilon_{lesion}(\theta) - \epsilon_{control}(\theta) \\ &\le \mathcal{R}_{seg} + |\epsilon_{lesion}(\theta) - \epsilon_{control}(\theta)|\end{aligned} \tag{B.1}$$

where $\epsilon_{lesion}(\theta)$ and $\epsilon_{control}(\theta)$ denote the expected tissue loss on the *lesion* and *control* domains, defined as:

$$\begin{aligned}\epsilon_{lesion}(\theta) &= \mathbb{E}_{\mathcal{D}_{lesion}}[\mathcal{L}^T(h_\theta(x^{T_1}), y^T))] \\ \epsilon_{control}(\theta) &= \mathbb{E}_{\mathcal{D}_{control}}[\mathcal{L}^T(h_\theta(x^{T_1}), y^T))]\end{aligned} \tag{B.2}$$

Let $\theta^* = \arg\min_{\theta\in\Theta} \epsilon_{lesion}(\theta) + \epsilon_{control}(\theta)$ be the parameters of the ideal (and unknown) segmenter that minimises the two expected tissue losses. Then let denote $\epsilon_{lesion}(\theta, \theta^*)$ and $\epsilon_{control}(\theta, \theta^*)$ the performance gap between the segmenter parametrised by θ and this ideal segmenter:

$$\begin{aligned}\epsilon_{lesion}(\theta,\theta^*) &= \mathbb{E}_{\mathcal{D}_{lesion}}[\mathcal{L}^T(h_\theta(x^{T_1}), h_{\theta^*}(x^{T_1}))] \\ \epsilon_{control}(\theta,\theta^*) &= \mathbb{E}_{\mathcal{D}_{control}}[\mathcal{L}^T(h_\theta(x^{T_1}), h_{\theta^*}(x^{T_1}))]\end{aligned} \tag{B.3}$$

Using the fact that the loss function satisfies the triangle inequality:

$$\begin{aligned}\epsilon_{lesion}(\theta) &\le \epsilon_{lesion}(\theta,\theta^*) + \epsilon_{lesion}(\theta^*) \\ \epsilon_{control}(\theta) &\le \epsilon_{control}(\theta,\theta^*) + \epsilon_{control}(\theta^*)\end{aligned} \tag{B.4}$$

Then, the performance gap between the two domains $|\epsilon_{lesion}(\theta) - \epsilon_{control}(\theta)|$ can be bounded as follows:

$$|\epsilon_{lesion}(\theta) - \epsilon_{control}(\theta)| \le |\epsilon_{lesion}(\theta,\theta^*) - \epsilon_{control}(\theta,\theta^*)| + \epsilon(\Theta) \tag{B.5}$$

where $\epsilon(\Theta)$ is the tissue expected loss of the ideal segmenter:

$$\epsilon(\Theta) = \epsilon_{lesion}(\theta^*) + \epsilon_{control}(\theta^*)$$

(B.5) can be found in Ben-David et al. (2010) in which the loss function is assumed to be the L_1 distance.

Given that $\epsilon(\Theta)$ is a constant w.r.t the network parameters θ, the goal of domain adaptation is to reduce the distribution discrepancy $d_{DA}(\theta)$ defined as:

$$d_{DA}(\theta) = |\epsilon_{lesion}(\theta,\theta^*) - \epsilon_{control}(\theta,\theta^*)| \tag{B.6}$$

Similarly to Long et al. (2018), we demonstrate that this discrepancy $d_{DA}(\theta)$ can be estimated using the discriminator accuracy.

Let denote $\mathcal{D}_l^\theta = (x^{T_1}, f_\theta(x^{T_1}))_{x^{T_1}\sim\mathcal{D}_{lesion}}$ and $\mathcal{D}_c^\theta = (x^{T_1}, f_\theta(x^{T_1}))_{x^{T_1}\sim\mathcal{D}_{control}}$ the proxies of the distributions $\mathcal{D}_{lesion}$ and $\mathcal{D}_{control}$. Then, the two performance gaps with the ideal segmenter can be re-written as:

$$\begin{aligned}\epsilon_{lesion}(\theta,\theta^*) &= \mathbb{E}_{(x,f)\sim\mathcal{D}_l^\theta}[\mathcal{L}^T(h_{\theta^*}(x^{T_1}), f)] \\ \epsilon_{lesion}(\theta,\theta^*) &= \mathbb{E}_{(x,f)\sim\mathcal{D}_l^\theta}[\mathcal{L}^T(h_{\theta^*}(x^{T_1}), f)]\end{aligned} \tag{B.7}$$

Let also define a difference hypothesis space Δ:

$$\Delta \triangleq \{\delta_{\theta'} : (x^{T_1}, f) \mapsto \mathcal{L}^T(h_{\theta'}(x^{T_1}), f)),\ \theta' \in \Theta\}$$

Moreover, we define the Δ-distance between the two distributions $\mathcal{D}_l^\theta$ and $\mathcal{D}_c^\theta$ as:

$$d_\Delta(\mathcal{D}_l^\theta, \mathcal{D}_c^\theta) \triangleq \sup_{\delta_{\theta'}\in\Delta} \left|\mathbb{E}_{\mathcal{D}_l^\theta}[\delta_{\theta'}(x^{T_1}, f)] - \mathbb{E}_{\mathcal{D}_c^\theta}[\delta_{\theta'}(x^{T_1}, f)]\right| \tag{B.8}$$

Finally, by combining (B.6), (B.7) and (B.8), we obtain the following upper bound for the distribution discrepancy $d_{DA}(\theta)$:

$$\begin{aligned}d_\Delta(\mathcal{D}_l^\theta, \mathcal{D}_c^\theta) &= \sup_{\delta_{\theta'}\in\Delta} \left|\mathbb{E}_{\mathcal{D}_l^\theta}[\delta_{\theta'}(x^{T_1}, f)] - \mathbb{E}_{\mathcal{D}_c^\theta}[\delta_{\theta'}(x^{T_1}, f)]\right| \\ &= \sup_{\theta'\in\Theta} \left|\mathbb{E}_{\mathcal{D}_l^\theta}[\mathcal{L}^T(h_{\theta'}(x^{T_1}), f)] - \mathbb{E}_{\mathcal{D}_c^\theta}[\mathcal{L}^T(h_{\theta'}(x^{T_1}), f)]\right| \\ &\ge \underbrace{\left|\mathbb{E}_{\mathcal{D}_l^\theta}[\mathcal{L}^T(h_{\theta^*}(x^{T_1}), f)] - \mathbb{E}_{\mathcal{D}_c^\theta}[\mathcal{L}^T(h_{\theta^*}(x^{T_1}), f)]\right|}_{=d_{DA}(\theta)}\end{aligned} \tag{B.9}$$

Finally, let $K > 0$ be the upper bound of the loss function $\mathcal{L}^T$ and $\mathcal{H}_\Phi^K$ denote the family of the discriminators multiplied by K:

$$\mathcal{H}_\Phi^K \triangleq \{KD_\phi,\ \phi \in \Phi\}$$

$\mathcal{H}_\Phi^K$ and the difference hypothesis space Δ are two continuous and K-bounded function classes. Similarly to Long et al. (2018), let's assume that the family of the discriminator $\mathcal{H}_\Phi^K$ is rich enough to contain the difference hypothesis space Δ. Given that a multilayer perceptrons that can fit any functions, this assumption is not unrealistic. Then, we show that the discriminator accuracy of the best discriminator is an upper bound of the Δ-distance:

$$\begin{aligned}d_\Delta(\mathcal{D}_l^\theta, \mathcal{D}_c^\theta) &\le K\sup_\phi \left|\mathbb{E}_{\mathcal{D}_l^\theta}[D(x^{T_1}, f)] - \mathbb{E}_{\mathcal{D}_c^\theta}[D(x^{T_1}, f)]\right| \\ &\le \underbrace{K\sup_\phi \left|\mathbb{E}_{\mathcal{D}_l^\theta}[D(x^{T_1}, f)] + \mathbb{E}_{\mathcal{D}_c^\theta}[1 - D(x^{T_1}, f)]\right|}_{=K\sup_\phi \mathcal{R}_{DA}(\phi,\theta)}\end{aligned} \tag{B.10}$$

Finally, by combining (B.1), (B.5), (B.9) (B.10), we obtain the following tractable upper bound for the expect segmentation loss:

$$\mathbb{E}_{\mathcal{D}_{lesion}}[\mathcal{L}(h_\theta(x), y)] \le \mathcal{R}_{seg} + K\sup_\phi \mathcal{R}_{DA}(\phi,\theta) + \epsilon(\Theta) \tag{B.11}$$

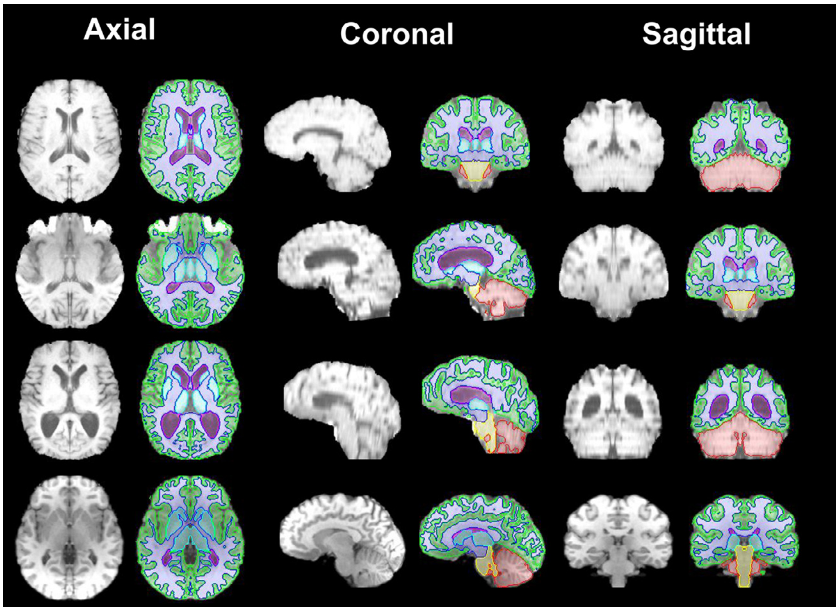

Fig. C.9. In order to synthesise a pseudo-healthy set of scans, we symmetrized the "healthy" hemisphere of brains from BraTS. GIF framework is then used to generate tissue ground truth. T_1scans are shown with the tissue segmentation.

Appendix C. Visualisation symmetrised brain scans

Fig. C.9 shows some examples of pseudo-healthy scans, with their tissue annotations, synthesised as described in 5.2.
